# The impact of evidence-based nursing leadership in healthcare settings: a mixed methods systematic review

**DOI:** 10.1186/s12912-024-02096-4

**Published:** 2024-07-03

**Authors:** Maritta Välimäki, Shuang Hu, Tella Lantta, Kirsi Hipp, Jaakko Varpula, Jiarui Chen, Gaoming Liu, Yao Tang, Wenjun Chen, Xianhong Li

**Affiliations:** 1 Department of Nursing Science, University of Turku, Turku, FI-20014 Finland; 2 School of Public Health, University of Helsinki, Helsinki, FI-00014 Finland; 3 Xiangya Nursing, School of Central South University, Changsha, 410013 China; 4 School of Health and Social Services, Häme University of Applied Sciences, Hämeenlinna, Finland; 5 Hunan Cancer Hospital, Changsha, 410008 China

**Keywords:** Evidence-based leadership, Health services administration, Organizational development, Quality in healthcare

## Abstract

**Background:**

The central component in impactful healthcare decisions is evidence. Understanding how nurse leaders use evidence in their own managerial decision making is still limited. This mixed methods systematic review aimed to examine how evidence is used to solve leadership problems and to describe the measured and perceived effects of evidence-based leadership on nurse leaders and their performance, organizational, and clinical outcomes.

**Methods:**

We included articles using any type of research design. We referred nurses, nurse managers or other nursing staff working in a healthcare context when they attempt to influence the behavior of individuals or a group in an organization using an evidence-based approach. Seven databases were searched until 11 November 2021. JBI Critical Appraisal Checklist for Quasi-experimental studies, JBI Critical Appraisal Checklist for Case Series, Mixed Methods Appraisal Tool were used to evaluate the Risk of bias in quasi-experimental studies, case series, mixed methods studies, respectively. The JBI approach to mixed methods systematic reviews was followed, and a parallel-results convergent approach to synthesis and integration was adopted.

**Results:**

Thirty-one publications were eligible for the analysis: case series (*n* = 27), mixed methods studies (*n* = 3) and quasi-experimental studies (*n* = 1). All studies were included regardless of methodological quality. Leadership problems were related to the implementation of knowledge into practice, the quality of nursing care and the resource availability. Organizational data was used in 27 studies to understand leadership problems, scientific evidence from literature was sought in 26 studies, and stakeholders’ views were explored in 24 studies. Perceived and measured effects of evidence-based leadership focused on nurses’ performance, organizational outcomes, and clinical outcomes. Economic data were not available.

**Conclusions:**

This is the first systematic review to examine how evidence is used to solve leadership problems and to describe its measured and perceived effects from different sites. Although a variety of perceptions and effects were identified on nurses’ performance as well as on organizational and clinical outcomes, available knowledge concerning evidence-based leadership is currently insufficient. Therefore, more high-quality research and clinical trial designs are still needed.

**Trail registration:**

The study was registered (PROSPERO CRD42021259624).

**Supplementary Information:**

The online version contains supplementary material available at 10.1186/s12912-024-02096-4.

## Background

Global health demands have set new roles for nurse leaders [1].Nurse leaders are referred to as nurses, nurse managers, or other nursing staff working in a healthcare context who attempt to influence the behavior of individuals or a group based on goals that are congruent with organizational goals [2]. They are seen as professionals “armed with data and evidence, and a commitment to mentorship and education”, and as a group in which “leaders innovate, transform, and achieve quality outcomes for patients, health care professionals, organizations, and communities” [3]. Effective leadership occurs when team members critically follow leaders and are motivated by a leader’s decisions based on the organization’s requests and targets [4]. On the other hand, problems caused by poor leadership may also occur, regarding staff relations, stress, sickness, or retention [5]. Therefore, leadership requires an understanding of different problems to be solved using synthesizing evidence from research, clinical expertise, and stakeholders’ preferences [6, 7]. If based on evidence, leadership decisions, also referred as leadership decision making [8], could ensure adequate staffing [7, 9] and to produce sufficient and cost-effective care [10]. However, nurse leaders still rely on their decision making on their personal [11] and professional experience [10] over research evidence, which can lead to deficiencies in the quality and safety of care delivery [12–14]. As all nurses should demonstrate leadership in their profession, their leadership competencies should be strengthened [15].

Evidence-informed decision-making, referred to as evidence appraisal and application, and evaluation of decisions [16], has been recognized as one of the core competencies for leaders [17, 18]. The role of evidence in nurse leaders’ managerial decision making has been promoted by public authorities [19–21]. Evidence-based management, another concept related to evidence-based leadership, has been used as the potential to improve healthcare services [22]. It can guide nursing leaders, in developing working conditions, staff retention, implementation practices, strategic planning, patient care, and success of leadership [13]. Collins and Holton [23] in their systematic review and meta-analysis examined 83 studies regarding leadership development interventions. They found that leadership training can result in significant improvement in participants’ skills, especially in knowledge level, although the training effects varied across studies. Cummings et al. [24] reviewed 100 papers (93 studies) and concluded that participation in leadership interventions had a positive impact on the development of a variety of leadership styles. Clavijo-Chamorro et al. [25] in their review of 11 studies focused on leadership-related factors that facilitate evidence implementation: teamwork, organizational structures, and transformational leadership. The role of nurse managers was to facilitate evidence-based practices by transforming contexts to motivate the staff and move toward a shared vision of change.

As far as we are aware, however, only a few systematic reviews have focused on evidence-based leadership or related concepts in the healthcare context aiming to analyse how nurse leaders themselves uses evidence in the decision-making process. Young [26] targeted definitions and acceptance of evidence-based management (EBMgt) in healthcare while Hasanpoor et al. [22] identified facilitators and barriers, sources of evidence used, and the role of evidence in the process of decision making. Both these reviews concluded that EBMgt was of great importance but used limitedly in healthcare settings due to a lack of time, a lack of research management activities, and policy constraints. A review by Williams [27] showed that the usage of evidence to support management in decision making is marginal due to a shortage of relevant evidence. Fraser [28] in their review further indicated that the potential evidence-based knowledge is not used in decision making by leaders as effectively as it could be. Non-use of evidence occurs and leaders base their decisions mainly on single studies, real-world evidence, and experts’ opinions [29]. Systematic reviews and meta-analyses rarely provide evidence of management-related interventions [30]. Tate et al. [31] concluded based on their systematic review and meta-analysis that the ability of nurse leaders to use and critically appraise research evidence may influence the way policy is enacted and how resources and staff are used to meet certain objectives set by policy. This can further influence staff and workforce outcomes. It is therefore important that nurse leaders have the capacity and motivation to use the strongest evidence available to effect change and guide their decision making [27].

Despite of a growing body of evidence, we found only one review focusing on the impact of evidence-based knowledge. Geert et al. [32] reviewed literature from 2007 to 2016 to understand the elements of design, delivery, and evaluation of leadership development interventions that are the most reliably linked to outcomes at the level of the individual and the organization, and that are of most benefit to patients. The authors concluded that it is possible to improve individual-level outcomes among leaders, such as knowledge, motivation, skills, and behavior change using evidence-based approaches. Some of the most effective interventions included, for example, interactive workshops, coaching, action learning, and mentoring. However, these authors found limited research evidence describing how nurse leaders themselves use evidence to support their managerial decisions in nursing and what the outcomes are.

To fill the knowledge gap and compliment to existing knowledgebase, in this mixed methods review we aimed to (1) examine what leadership problems nurse leaders solve using an evidence-based approach and (2) how they use evidence to solve these problems. We also explored (3) the measured and (4) perceived effects of the evidence-based leadership approach in healthcare settings. Both qualitative and quantitative components of the effects of evidence-based leadership were examined to provide greater insights into the available literature [33]. Together with the evidence-based leadership approach, and its impact on nursing [34, 35], this knowledge gained in this review can be used to inform clinical policy or organizational decisions [33]. The study is registered (PROSPERO CRD42021259624). The methods used in this review were specified in advance and documented in a priori in a published protocol [36]. Key terms of the review and the search terms are defined in Table 1 (population, intervention, comparison, outcomes, context, other).

## Methods

### Design

In this review, we used a mixed methods approach [37]. A mixed methods systematic review was selected as this approach has the potential to produce direct relevance to policy makers and practitioners [38]. Johnson and Onwuegbuzie [39] have defined mixed methods research as “the class of research in which the researcher mixes or combines quantitative and qualitative research techniques, methods, approaches, concepts or language into a single study.” Therefore, we combined quantitative and narrative analysis to appraise and synthesize empirical evidence, and we held them as equally important in informing clinical policy or organizational decisions [34]. In this review, a comprehensive synthesis of quantitative and qualitative data was performed first and then discussed in discussion part (parallel-results convergent design) [40]. We hoped that different type of analysis approaches could complement each other and deeper picture of the topic in line with our research questions could be gained [34].

### Inclusion and exclusion criteria

Inclusion and exclusion criteria of the study are described in Table 1.

**Table 1:** Inclusion and exclusion criteria for the study

Criteria	Description of inclusion and exclusion criteria
Population	Inclusion: Articles dealt with nurses, nurse managers or other nursing staff working in a healthcare context; they need to have an official or unofficial managerial role in the organization as leadership occurs whenever a person attempts to influence the behavior of individuals or a group based on personal goals or goals of others that are congruent with organizational goals [2]. Exclusion: If nurses were not a clear majority (50% or more) in the sample.
Intervention	Inclusion: One or more of the five steps of the evidence-based practice process were evident [41, 42]: (1) a nurse leader identifies a problem to be solved related to their leadership practice, (2) organizational evidence or data about the leadership problem or issue are collected and analyzed to check for relevance and validity, and the problem is restated, reformulated or made more specific, (3) scientific evidence from published research about the leadership problem is identified and critically appraised, (4) the views of stakeholders (patients, clinicians, family members, etc.) are considered, and (5) all sources of information are critically appraised [33].
Comparison	If an included study would use a randomized trial design, we would include another type of intervention as a comparison group.
Outcomes	Studies described any outcomes related to the individual or group performance of nurses or nurse managers in terms of leadership skills (e.g., communication skills), organizational outcomes (e.g., work environment, costs), healthcare provider outcomes (e.g., job satisfaction) or clinical outcomes (e.g., patient quality of life, treatment satisfaction).
Context	Inclusion: We use the term leadership to refer to the process of when a person attempts to influence the behavior of individuals or a group in an organization for any reason [2]. Evidence-based leadership occurs when the behavior of individuals or a group is affected using an evidence-based approach in a healthcare context. We propose that evidence-based leadership is analogous to EBMgt [33, 43].
Other	Inclusion: Any type of research design if they included leadership as a research topic as well as any component of an evidence-based leadership approach; peer-reviewed, published full-text articles or conference abstracts/proceedings with no language restriction. Exclusion: Theoretical papers, statistical reviews, books and book chapters, letters, dissertations, editorials, study protocols.

### Search strategy

A three-step search strategy was utilized. First, an initial limited search with #MEDLINE was undertaken, followed by analysis of the words used in the title, abstract, and the article’s key index terms. Second, the search strategy, including identified keywords and index terms, was adapted for each included data base and a second search was undertaken on 11 November 2021. The full search strategy for each database is described in Additional file 1. Third, the reference list of all studies included in the review were screened for additional studies. No year limits or language restrictions were used.

### Information sources

The database search included the following: CINAHL (EBSCO), Cochrane Library (academic database for medicine and health science and nursing), Embase (Elsevier), PsycINFO (EBSCO), PubMed (MEDLINE), Scopus (Elsevier) and Web of Science (academic database across all scientific and technical disciplines, ranging from medicine and social sciences to arts and humanities). These databases were selected as they represent typical databases in health care context. Subject headings from each of the databases were included in the search strategies. Boolean operators ‘AND’ and ‘OR’ were used to combine the search terms. An information specialist from the University of Turku Library was consulted in the formation of the search strategies.

### Study selection

All identified citations were collated and uploaded into Covidence software (Covidence systematic review software, Veritas Health Innovation, Melbourne, Australia www.covidence.org), and duplicates were removed by the software. Titles and abstracts were screened and assessed against the inclusion criteria independently by two reviewers out of four, and any discrepancies were resolved by the third reviewer (MV, KH, TL, WC). Studies meeting the inclusion criteria were retrieved in full and archived in Covidence. Access to one full-text article was lacking: the authors for one study were contacted about the missing full text, but no full text was received. All remaining hits of the included studies were retrieved and assessed independently against the inclusion criteria by two independent reviewers of four (MV, KH, TL, WC). Studies that did not meet the inclusion criteria were excluded, and the reasons for exclusion were recorded in Covidence. Any disagreements that arose between the reviewers were resolved through discussions with XL.

### Assessment of methodological quality

Eligible studies were critically appraised by two independent reviewers (YT, SH). Standardized critical appraisal instruments based on the study design were used. First, quasi-experimental studies were assessed using the JBI Critical Appraisal Checklist for Quasi-experimental studies [44]. Second, case series were assessed using the JBI Critical Appraisal Checklist for Case Series [45]. Third, mixed methods studies were appraised using the Mixed Methods Appraisal Tool [46].

To increase inter-reviewer reliability, the review agreement was calculated (SH) [47]. A kappa greater than 0.8 was considered to represent a high level of agreement (0–0.1). In our data, the agreement was 0.75. Discrepancies raised between two reviewers were resolved through discussion and modifications and confirmed by XL. As an outcome, studies that met the inclusion criteria were proceeded to critical appraisal and assessed as suitable for inclusion in the review. The scores for each item and overall critical appraisal scores were presented.

### Data extraction

For data extraction, specific tables were created. First, study characteristics (author(s), year, country, design, number of participants, setting) were extracted by two authors independently (JC, MV) and reviewed by TL. Second, descriptions of the interventions were extracted by two reviewers (JV, JC) using the structure of the TIDIeR (Template for Intervention Description and Replication) checklist (brief name, the goal of the intervention, material and procedure, models of delivery and location, dose, modification, adherence and fidelity) [48]. The extractions were confirmed (MV).

Third, due to a lack of effectiveness data and a wide heterogeneity between study designs and presentation of outcomes, no attempt was made to pool the quantitative data statistically; the findings of the quantitative data were presented in narrative form only [44]. The separate data extraction tables for each research question were designed specifically for this study. For both qualitative (and a qualitative component of mixed-method studies) and quantitative studies, the data were extracted and tabulated into text format according to preplanned research questions [36]. To test the quality of the tables and the data extraction process, three authors independently extracted the data from the first five studies (in alphabetical order). After that, the authors came together to share and determine whether their approaches of the data extraction were consistent with each other’s output and whether the content of each table was in line with research question. No reason was found to modify the data extraction tables or planned process. After a consensus of the data extraction process was reached, the data were extracted in pairs by independent reviewers (WC, TY, SH, GL). Any disagreements that arose between the reviewers were resolved through discussion and with a third reviewer (MV).

### Data analysis

We were not able to conduct a meta-analysis due to a lack of effectiveness data based on clinical trials. Instead, we used inductive thematic analysis with constant comparison to answer the research question [46, 49] using tabulated primary data from qualitative and quantitative studies as reported by the original authors in narrative form only [47]. In addition, the qualitizing process was used to transform quantitative data to qualitative data; this helped us to convert the whole data into themes and categories. After that we used the thematic analysis for the narrative data as follows. First, the text was carefully read, line by line, to reveal topics answering each specific review question (MV). Second, the data coding was conducted, and the themes in the data were formed by data categorization. The process of deriving the themes was inductive based on constant comparison [49]. The results of thematic analysis and data categorization was first described in narrative format and then the total number of studies was calculated where the specific category was identified (%).

### Stakeholder involvement

The method of reporting stakeholders’ involvement follows the key components by [50]: (1) people involved, (2) geographical location, (3) how people were recruited, (4) format of involvement, (5) amount of involvement, (6) ethical approval, (7) financial compensation, and (8) methods for reporting involvement.

In our review, stakeholder involvement targeted nurses and nurse leader in China. Nurse Directors of two hospitals recommended potential participants who received a personal invitation letter from researchers to participate in a discussion meeting. Stakeholders’ participation was based on their own free will. Due to COVID-19, one online meeting (1 h) was organized (25 May 2022). Eleven participants joined the meeting. Ethical approval was not applied and no financial compensation was offered. At the end of the meeting, experiences of stakeholders’ involvement were explored.

The meeting started with an introductory presentation with power points. The rationale, methods, and preliminary review results were shared with the participants [51].The meeting continued with general questions for the participants: (1) Are you aware of the concepts of evidence-based practice or evidence-based leadership?; (2) How important is it to use evidence to support decisions among nurse leaders?; (3) How is the evidence-based approach used in hospital settings?; and (4) What type of evidence is currently used to support nurse leaders’ decision making (e.g. scientific literature, organizational data, stakeholder views)?

Two people took notes on the course and content of the conversation. The notes were later transcripted in verbatim, and the key points of the discussions were summarised. Although answers offered by the stakeholders were very short, the information was useful to validate the preliminary content of the results, add the rigorousness of the review, and obtain additional perspectives. A recommendation of the stakeholders was combined in the Discussion part of this review increasing the applicability of the review in the real world [50]. At the end of the discussion, the value of stakeholders’ involvement was asked. Participants shared that the experience of participating was unique and the topic of discussion was challenging. Two authors of the review group further represented stakeholders by working together with the research team throughout the review study.

## Results

### Search results

**Fig. 1:**
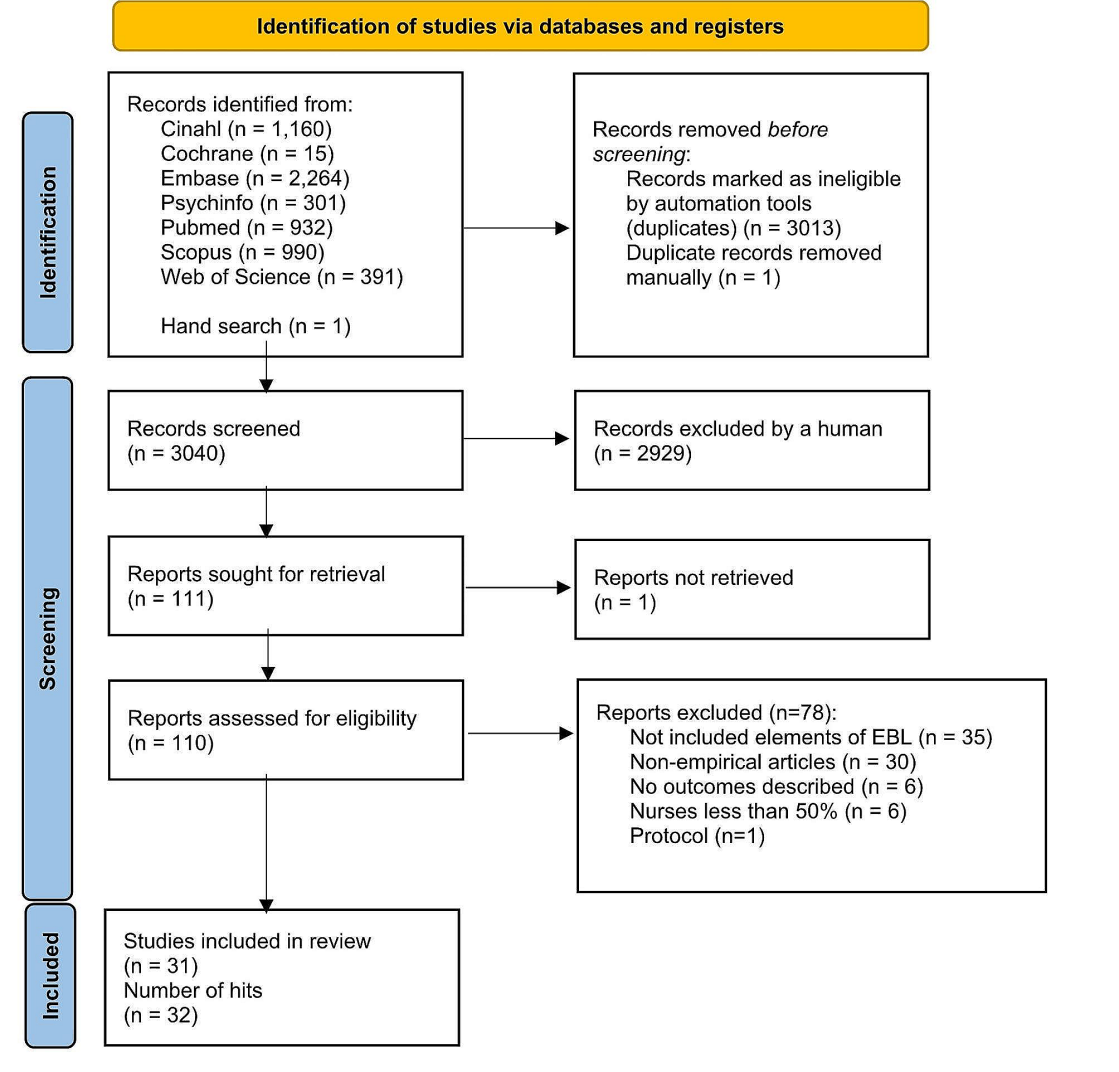
Search results and study selection and inclusion process [52]

From seven different electronic databases, 6053 citations were identified as being potentially relevant to the review. Then, 3133 duplicates were removed by an automation tool (Covidence: www.covidence.org), and one was removed manually. The titles and abstracts of 3040 of citations were reviewed, and a total of 110 full texts were included (one extra citation was found on the reference list but later excluded). Based on the eligibility criteria, 31 studies (32 hits) were critically appraised and deemed suitable for inclusion in the review. The search results and selection process are presented in the PRISMA [52] flow diagram Fig. 1. The full list of references for included studies can be find in Additional file 2. To avoid confusion between articles of the reference list and studies included in the analysis, the studies included in the review are referred inside the article using the reference number of each study (e.g. ref 1, ref 2).

### Characteristics of included studies

The studies had multiple purposes, aiming to develop practice, implement a new approach, improve quality, or to develop a model. The 31 studies (across 32 hits) were case series studies (*n* = 27), mixed methods studies (*n* = 3) and a quasi-experimental study (*n* = 1). All studies were published between the years 2004 and 2021. The highest number of papers was published in year 2020.

Table 2 describes the characteristics of included studies and Additional file 3 offers a narrative description of the studies.

**Table 2:** Characteristics of included studies

Author(s) (year) (Ref #)	Country	Design Study type	Number and type of participants	Setting
Alleyne & Jumaa (2007) (Ref 1)	UK	Case series Action research and case study with multi-method data collection	6 district nurses 2 doctoral candidates	Hospital
Busbee et al. (2020 a, b) (Ref 2)	US	Case series Practice development project	14 facility leads	Hospital
Cullen & Titler (2004) (Ref 3)	US	Case series Multimethod study	6 nurses (quantitative data) 6 nurses, 5 managers (focus groups)	Hospital
Davidson & Brown (2014) (Ref 4)	US	Case series Implementation study	15 nurses	Hospital
DeLeskey (2009) (Ref 5)	US	Case series Implementation study	79 patients	Hospital
Galiano et al. (2020) (Ref 6)	Chile	Case series Implementation study	510 nurses	Hospital
Gifford et al. (2011) (Ref 7)	Canada	Case series Implementation study	15 nurse managers and clinical leaders	Home and community healthcare organization
Gifford et al. (2013) (Ref 8)	Canada	Mixed methods study Pilot study	NA Clinical and management leadership teams	Home healthcare organization
Gifford et al. (2014) (Ref 9)	Canada	Mixed methods study Implementation study	Management and clinical leaders; preintervention *n* = 32, postintervention *n* = 17, surveys/interviews *n* = 15	Home healthcare organization
Hester et al. (2016) (Ref 10)	US	Case series Implementation study	NA Staff nurses	Hospital
Hoke et al. (2016) (Ref 11)	US	Case series Implementation study	85 patient records; pre-intervention *n* = 42, post-intervention *n* = 43	Hospital
Hsieh et al. (2016) (Ref 12)	China (Taiwan)	Case series Implementation study	22 nurses	Hospital
Kidd et al. (2020) (Ref 13)	US	Case series Development study	177 resident nurses	Hospital
Kneflin et al. (2016) (Ref 14)	US	Case series Positioning paper	NA Nurses, nurse leaders	Hospital
Laws et al. (2013) (Ref 15)	US	Case series Implementation study	NA Nurse leaders	Hospital
McAllen et al. (2018) (Ref 16)	US	Qase series Quality improvement	67 nurses	Hospital
McDonough & Pemberton (2013) (Ref 17)	US	Case series Implementation study	NA Emergency department staff	Hospital
McFarlan et al. (2019) (Ref 18)	US	Case series Practice development	75 nurses 25 paramedics 6 patient-care technicians	Hospital
McKinley et al. (2007) (Ref 19)	Australia	Case series Quality management initiative program	NA In-hospital patient population	Hospital
Ostaszkiewicz et al. (2021) (Ref 20)	Australia	Case series Multimethod study	22 nurses	Aged care homes
Parchment & Stinson (2020) (Ref 21)	US	Case series Improvement project	NA Inter-disciplinary groups, the local nonprofit community, state agencies	NA
Britt Pipe (2007) (Ref 22)	US	Case series Practice development	NA Nurses	Hospital
Robbins et al. (2017) (Ref 23)	US	Case series Implementation project	110 nurses	Hospital
Salvador & Howell (2010) (Ref 24)	Canada	Case series Model development	NA Nurses	Hospital
Stacey et al. (2019) (Ref 25)	Canada	Case series Comparative case study	489 nurses	Nursing agencies
Sving et al. (2020) (Ref 26)	Sweden	Mixed methods study Sequential mixed method approach	259 patients (quantitative) 32 (qualitative) nurses	Hospital
Tafelmeyer et al. (2017) (Ref 27)	US	Case series Pre-post assessment	NA Nurses	Hospital
Thomas & Donohue-Porter (2012) (Ref 28)	US	Case series Implementation project	6 hospitals	Hospital
Thomas et al. (2020) (Ref 29)	US	Case series Implementation project	NA Nurses	Hospital
Van Orne (2021) (Ref 30)	US	Case series Quality improvement project	NA Nurses	Hospital
Yurumezoglu & Kocaman (2012) (Ref 31)	Turkey	Quasi-experimental study A pilot study	158 nurses 1st and 2nd follow-up *n* = 58	Hospital

NA = No information available

### Methodological quality assessment

#### Quasi-experimental studies

We had one quasi-experimental study (ref 31). All questions in the critical appraisal tool were applicable. The total score of the study was 8 (out of a possible 9). Only one response of the tool was ‘no’ because no control group was used in the study (see Additional file 4 for the critical appraisal of included studies).

*Case series studies*. A case series study is typically defined as a collection of subjects with common characteristics. The studies do not include a comparison group and are often based on prevalent cases and on a sample of convenience [53]. Munn et al. [45] further claim that case series are best described as observational studies, lacking experimental and randomized characteristics, being descriptive studies, without a control or comparator group. Out of 27 case series studies included in our review, the critical appraisal scores varied from 1 to 9. Five references were conference abstracts with empirical study results, which were scored from 1 to 3. Full reports of these studies were searched in electronic databases but not found. Critical appraisal scores for the remaining 22 studies ranged from 1 to 9 out of a possible score of 10. One question (Q3) was not applicable to 13 studies: “Were valid methods used for identification of the condition for all participants included in the case series?” Only two studies had clearly reported the demographic of the participants in the study (Q6). Twenty studies met Criteria 8 (“Were the outcomes or follow-up results of cases clearly reported?”) and 18 studies met Criteria 7 (“Q7: Was there clear reporting of clinical information of the participants?”) (see Additional file 4 for the critical appraisal of included studies).

#### Mixed-methods studies

Mixed-methods studies involve a combination of qualitative and quantitative methods. This is a common design and includes convergent design, sequential explanatory design, and sequential exploratory design [46]. There were three mixed-methods studies. The critical appraisal scores for the three studies ranged from 60 to 100% out of a possible 100%. Two studies met all the criteria, while one study fulfilled 60% of the scored criteria due to a lack of information to understand the relevance of the sampling strategy well enough to address the research question (Q4.1) or to determine whether the risk of nonresponse bias was low (Q4.4) (see Additional file 4 for the critical appraisal of included studies).

### Intervention or program components

The intervention of program components were categorized and described using the TiDier checklist: name and goal, theory or background, material, procedure, provider, models of delivery, location, dose, modification, and adherence and fidelity [48]. A description of intervention in each study is described in Additional file 5 and a narrative description in Additional file 6.

### Leadership problems

In line with the inclusion criteria, data for the leadership problems were categorized in all 31 included studies (see Additional file 7 for leadership problems). Three types of leadership problems were identified: implementation of knowledge into practice, the quality of clinical care, and resources in nursing care. A narrative summary of the results is reported below.

#### **Implementing knowledge into practice**

Eleven studies (35%) aimed to solve leadership problems related to implementation of knowledge into practice. Studies showed how to support nurses in evidence-based implementation (EBP) (ref 3, ref 5), how to engage nurses in using evidence in practice (ref 4), how to convey the importance of EBP (ref 22) or how to change practice (ref 4). Other problems were how to facilitate nurses to use guideline recommendations (ref 7) and how nurses can make evidence-informed decisions (ref 8). General concerns also included the linkage between theory and practice (ref 1) as well as how to implement the EBP model in practice (ref 6). In addition, studies were motivated by the need for revisions or updates of protocols to improve clinical practice (ref 10) as well as the need to standardize nursing activities (ref 11, ref 14).

#### **The quality of the care**

Thirteen (42%) focused on solving problems related to the quality of clinical care. In these studies, a high number of catheter infections led a lack of achievement of organizational goals (ref 2, ref 9). A need to reduce patient symptoms in stem cell transplant patients undergoing high-dose chemotherapy (ref 24) was also one of the problems to be solved. In addition, the projects focused on how to prevent pressure ulcers (ref 26, ref 29), how to enhance the quality of cancer treatment (ref 25) and how to reduce the need for invasive constipation treatment (ref 30). Concerns about patient safety (ref 15), high fall rates (ref 16, ref 19), dissatisfaction of patients (ref 16, ref 18) and nurses (ref 16, ref 30) were also problems that had initiated the projects. Studies addressed concerns about how to promote good contingency care in residential aged care homes (ref 20) and about how to increase recognition of human trafficking problems in healthcare (ref 21).

#### **Resources in nursing care**

Nurse leaders identified problems in their resources, especially in staffing problems. These problems were identified in seven studies (23%), which involved concerns about how to prevent nurses from leaving the job (ref 31), how to ensure appropriate recruitment, staffing and retaining of nurses (ref 13) and how to decrease nurses’ burden and time spent on nursing activities (ref 12). Leadership turnover was also reported as a source of dissatisfaction (ref 17); studies addressed a lack of structured transition and training programs, which led to turnover (ref 23), as well as how to improve intershift handoff among nurses (ref 28). Optimal design for new hospitals was also examined (ref 27).

### Main features of evidence-based leadership

Out of 31 studies, 17 (55%) included all four domains of an evidence-based leadership approach, and four studies (13%) included evidence of critical appraisal of the results (see Additional file 8 for the main features of evidence-based Leadership) (ref 11, ref 14, ref 23, ref 27).

#### ***Organizational evidence***

Twenty-seven studies (87%) reported how organizational evidence was collected and used to solve leadership problems (ref 2). Retrospective chart reviews (ref 5), a review of the extent of specific incidents (ref 19), and chart auditing (ref 7, ref 25) were conducted. A gap between guideline recommendations and actual care was identified using organizational data (ref 7) while the percentage of nurses’ working time spent on patient care was analyzed using an electronic charting system (ref 12). Internal data (ref 22), institutional data, and programming metrics were also analyzed to understand the development of the nurse workforce (ref 13).

Surveys (ref 3, ref 25), interviews (ref 3, ref 25) and group reviews (ref 18) were used to better understand the leadership problem to be solved. Employee opinion surveys on leadership (ref 17), a nurse satisfaction survey (ref 30) and a variety of reporting templates were used for the data collection (ref 28) reported. Sometimes, leadership problems were identified by evidence facilitators or a PI’s team who worked with staff members (ref 15, ref 17). Problems in clinical practice were also identified by the Nursing Professional Council (ref 14), managers (ref 26) or nurses themselves (ref 24). Current practices were reviewed (ref 29) and a gap analysis was conducted (ref 4, ref 16, ref 23) together with SWOT analysis (ref 16). In addition, hospital mission and vision statements, research culture established and the proportion of nursing alumni with formal EBP training were analyzed (ref 5). On the other hand, it was stated that no systematic hospital-specific sources of data regarding job satisfaction or organizational commitment were used (ref 31). In addition, statements of organizational analysis were used on a general level only (ref 1).

#### ***Scientific evidence identified***

Twenty-six studies (84%) reported the use of scientific evidence in their evidence-based leadership processes. A literature search was conducted (ref 21) and questions, PICO, and keywords were identified (ref 4) in collaboration with a librarian. Electronic databases, including PubMed (ref 14, ref 31), Cochrane, and EMBASE (ref 31) were searched. Galiano (ref 6) used Wiley Online Library, Elsevier, CINAHL, Health Source: Nursing/Academic Edition, PubMed, and the Cochrane Library while Hoke (ref 11) conducted an electronic search using CINAHL and PubMed to retrieve articles.

Identified journals were reviewed manually (ref 31). The findings were summarized using ‘elevator speech’ (ref 4). In a study by Gifford et al. (ref 9) evidence facilitators worked with participants to access, appraise, and adapt the research evidence to the organizational context. Ostaszkiewicz (ref 20) conducted a scoping review of literature and identified and reviewed frameworks and policy documents about the topic and the quality standards. Further, a team of nursing administrators, directors, staff nurses, and a patient representative reviewed the literature and made recommendations for practice changes.

Clinical practice guidelines were also used to offer scientific evidence (ref 7, ref 19). Evidence was further retrieved from a combination of nursing policies, guidelines, journal articles, and textbooks (ref 12) as well as from published guidelines and literature (ref 13). Internal evidence, professional practice knowledge, relevant theories and models were synthesized (ref 24) while other study (ref 25) reviewed individual studies, synthesized with systematic reviews or clinical practice guidelines. The team reviewed the research evidence (ref 3, ref 15) or conducted a literature review (ref 22, ref 28, ref 29), a literature search (ref 27), a systematic review (ref 23), a review of the literature (ref 30) or ‘the scholarly literature was reviewed’ (ref 18). In addition, ‘an extensive literature review of evidence-based best practices was carried out’ (ref 10). However, detailed description how the review was conducted was lacking.

#### ***Views of stakeholders***

A total of 24 studies (77%) reported methods for how the views of stakeholders, i.e., professionals or experts, were considered. Support to run this study was received from nursing leadership and multidisciplinary teams (ref 29). Experts and stakeholders joined the study team in some cases (ref 25, ref 30), and in other studies, their opinions were sought to facilitate project success (ref 3). Sometimes a steering committee was formed by a Chief Nursing Officer and Clinical Practice Specialists (ref 2). More specifically, stakeholders’ views were considered using interviews, workshops and follow-up teleconferences (ref 7). The literature review was discussed with colleagues (ref 11), and feedback and support from physicians as well as the consensus of staff were sought (ref 16).

A summary of the project findings and suggestions for the studies were discussed at 90-minute weekly meetings by 11 charge nurses. Nurse executive directors were consulted over a 10-week period (ref 31). An implementation team (nurse, dietician, physiotherapist, occupational therapist) was formed to support the implementation of evidence-based prevention measures (ref 26). Stakeholders volunteered to join in the pilot implementation (ref 28) or a stakeholder team met to determine the best strategy for change management, shortcomings in evidence-based criteria were discussed, and strategies to address those areas were planned (ref 5). Nursing leaders, staff members (ref 22), ‘process owners (ref 18) and program team members (ref 18, ref 19, ref 24) met regularly to discuss the problems. Critical input was sought from clinical educators, physicians, nutritionists, pharmacists, and nurse managers (ref 24). The unit director and senior nursing staff reviewed the contents of the product, and the final version of clinical pathways were reviewed and approved by the Quality Control Commission of the Nursing Department (ref 12). In addition, two co-design workshops with 18 residential aged care stakeholders were organized to explore their perspectives about factors to include in a model prototype (ref 20). Further, an agreement of stakeholders in implementing continuous quality services within an open relationship was conducted (ref 1).

#### ***Critical appraisal***

In five studies (16%), a critical appraisal targeting the literature search was carried out. The appraisals were conducted by interns and teams who critiqued the evidence (ref 4). In Hoke’s study, four areas that had emerged in the literature were critically reviewed (ref 11). Other methods were to ‘critically appraise the search results’ (ref 14). Journal club team meetings (ref 23) were organized to grade the level and quality of evidence and the team ‘critically appraised relevant evidence’ (ref 27). On the other hand, the studies lacked details of how the appraisals were done in each study.

### The perceived effects of evidence-based leadership

#### ***Perceived effects of evidence-based leadership on nurses’ performance***

Eleven studies (35%) described perceived effects of evidence-based leadership on nurses’ performance (see Additional file 9 for perceived effects of evidence-based leadership), which were categorized in four groups: awareness and knowledge, competence, ability to understand patients’ needs, and engagement. First, regarding ‘awareness and knowledge’, different projects provided nurses with new learning opportunities (ref 3). Staff’s knowledge (ref 20, ref 28), skills, and education levels improved (ref 20), as did nurses’ knowledge comprehension (ref 21). Second, interventions and approaches focusing on management and leadership positively influenced participants’ competence level to improve the quality of services. Their confidence level (ref 1) and motivation to change practice increased, self-esteem improved, and they were more positive and enthusiastic in their work (ref 22). Third, some nurses were relieved that they had learned to better handle patients’ needs (ref 25). For example, a systematic work approach increased nurses’ awareness of the patients who were at risk of developing health problems (ref 26). And last, nurse leaders were more engaged with staff, encouraging them to adopt the new practices and recognizing their efforts to change (ref 8).

#### ***Perceived effects on organizational outcomes***

Nine studies (29%) described the perceived effects of evidence-based leadership on organizational outcomes (see Additional file 9 for perceived effects of evidence-based leadership). These were categorized into three groups: use of resources, staff commitment, and team effort. First, more appropriate use of resources was reported (ref 15, ref 20), and working time was more efficiently used (ref 16). In generally, a structured approach made implementing change more manageable (ref 1). On the other hand, in the beginning of the change process, the feedback from nurses was unfavorable, and they experienced discomfort in the new work style (ref 29). New approaches were also perceived as time consuming (ref 3). Second, nurse leaders believed that fewer nursing staff than expected left the organization over the course of the study (ref 31). Third, the project helped staff in their efforts to make changes, and it validated the importance of working as a team (ref 7). Collaboration and support between the nurses increased (ref 26). On the other hand, new work style caused challenges in teamwork (ref 3).

#### ***Perceived effects on clinical outcomes***

Five studies (16%) reported the perceived effects of evidence-based leadership on clinical outcomes (see Additional file 9 for perceived effects of evidence-based leadership), which were categorized in two groups: general patient outcomes and specific clinical outcomes. First, in general, the project assisted in connecting the guideline recommendations and patient outcomes (ref 7). The project was good for the patients in general, and especially to improve patient safety (ref 16). On the other hand, some nurses thought that the new working style did not work at all for patients (ref 28). Second, the new approach used assisted in optimizing patients’ clinical problems and person-centered care (ref 20). Bowel management, for example, received very good feedback (ref 30).

### The measured effects of evidence-based leadership

#### ***The measured effects on nurses’ performance***

Data were obtained from 20 studies (65%) (see Additional file 10 for measured effects of evidence-based leadership) and categorized nurse performance outcomes for three groups: awareness and knowledge, engagement, and satisfaction. First, six studies (19%) measured the awareness and knowledge levels of participants. Internship for staff nurses was beneficial to help participants to understand the process for using evidence-based practice and to grow professionally, to stimulate for innovative thinking, to give knowledge needed to use evidence-based practice to answer clinical questions, and to make possible to complete an evidence-based practice project (ref 3). Regarding implementation program of evidence-based practice, those with formal EBP training showed an improvement in knowledge, attitude, confidence, awareness and application after intervention (ref 3, ref 11, ref 20, ref 23, ref 25). On the contrary, in other study, attitude towards EBP remained stable (*p* = 0.543). and those who applied EBP decreased although no significant differences over the years (*p* = 0.879) (ref 6).

Second, 10 studies (35%) described nurses’ engagement to new practices (ref 5, ref 6, ref 7, ref 10, ref 16, ref 17, ref 18, ref 21, ref 25, ref 27). 9 studies (29%) studies reported that there was an improvement of compliance level of participants (ref 6, ref 7, ref 10, ref 16, ref 17, ref 18, ref 21, ref 25, ref 27). On the contrary, in DeLeskey’s (ref 5) study, although improvement was found in post-operative nausea and vomiting’s (PONV) risk factors documented’ (2.5–63%), and ’risk factors communicated among anaesthesia and surgical staff’ (0–62%), the improvement did not achieve the goal. The reason was a limited improvement was analysed. It was noted that only those patients who had been seen by the pre-admission testing nurse had risk assessments completed. Appropriate treatment/prophylaxis increased from 69 to 77%, and from 30 to 49%; routine assessment for PONV/rescue treatment 97% and 100% was both at 100% following the project. The results were discussed with staff but further reasons for a lack of engagement in nursing care was not reported.

And third, six studies (19%) reported nurses’ satisfaction with project outcomes. The study results showed that using evidence in managerial decisions improved nurses’ satisfaction and attitudes toward their organization (*P* < 0.05) (ref 31). Nurses’ overall job satisfaction improved as well (ref 17). Nurses’ satisfaction with usability of the electronic charting system significantly improved after introduction of the intervention (ref 12). In handoff project in seven hospitals, improvement was reported in all satisfaction indicators used in the study although improvement level varied in different units (ref 28). In addition, positive changes were reported in nurses’ ability to autonomously perform their job (“How satisfied are you with the tools and resources available for you treat and prevent patient constipation?” (54%, *n* = 17 vs. 92%, *n* = 35, *p* < 0.001) (ref 30).

#### ***The measured effects on organizational outcomes***

Thirteen studies (42%) described the effects of a project on organizational outcomes (see Additional file 10 for measured effects of evidence-based leadership), which were categorized in two groups: staff compliance, and changes in practices. First, studies reported improved organizational outcomes due to staff better compliance in care (ref 4, ref 13, ref 17, ref 23, ref 27, ref 31). Second, changes in organization practices were also described (ref 11) like changes in patient documentation (ref 12, ref 21). Van Orne (ref 30) found a statistically significant reduction in the average rate of invasive medication administration between pre-intervention and post-intervention (*p* = 0.01). Salvador (ref 24) also reported an improvement in a proactive approach to mucositis prevention with an evidence-based oral care guide. On the contrary, concerns were also raised such as not enough time for new bedside report (ref 16) or a lack of improvement of assessment of diabetic ulcer (ref 8).

#### ***The measured effects on clinical outcomes***

A variety of improvements in clinical outcomes were reported (see Additional file 10 for measured effects of evidence-based leadership): improvement in patient clinical status and satisfaction level. First, a variety of improvement in patient clinical status was reported. improvement in Incidence of CAUTI decreased 27.8% between 2015 and 2019 (ref 2) while a patient-centered quality improvement project reduced CAUTI rates to 0 (ref 10). A significant decrease in transmission rate of MRSA transmission was also reported (ref 27) and in other study incidences of CLABSIs dropped following of CHG bathing (ref 14). Further, it was possible to decrease patient nausea from 18 to 5% and vomiting to 0% (ref 5) while the percentage of patients who left the hospital without being seen was below 2% after the project (ref 17). In addition, a significant reduction in the prevalence of pressure ulcers was found (ref 26, ref 29) and a significant reduction of mucositis severity/distress was achieved (ref 24). Patient falls rate decreased (ref 15, ref 16, ref 19, ref 27).

Second, patient satisfaction level after project implementation improved (ref 28). The scale assessing healthcare providers by consumers showed improvement, but the changes were not statistically significant. Improvement in an emergency department leadership model and in methods of communication with patients improved patient satisfaction scores by 600% (ref 17). In addition, new evidence-based unit improved patient experiences about the unit although not all items improved significantly (ref 18).

### Stakeholder involvement in the mixed-method review

To ensure stakeholders’ involvement in the review, the real-world relevance of our research [53], achieve a higher level of meaning in our review results, and gain new perspectives on our preliminary findings [50], a meeting with 11 stakeholders was organized. First, we asked if participants were aware of the concepts of evidence-based practice or evidence-based leadership. Responses revealed that participants were familiar with the concept of evidence-based practice, but the topic of evidence-based leadership was totally new. Examples of nurses and nurse leaders’ responses are as follows: “I have heard a concept of evidence-based practice but never a concept of evidence-based leadership.” Another participant described: “I have heard it [evidence-based leadership] but I do not understand what it means.”

Second, as stakeholder involvement is beneficial to the relevance and impact of health research [54], we asked how important evidence is to them in supporting decisions in health care services. One participant described as follows: “Using evidence in decisions is crucial to the wards and also to the entire hospital.” Third, we asked how the evidence-based approach is used in hospital settings. Participants expressed that literature is commonly used to solve clinical problems in patient care but not to solve leadership problems. “In [patient] medication and care, clinical guidelines are regularly used. However, I am aware only a few cases where evidence has been sought to solve leadership problems.”

And last, we asked what type of evidence is currently used to support nurse leaders’ decision making (e.g. scientific literature, organizational data, stakeholder views)? The participants were aware that different types of information were collected in their organization on a daily basis (e.g. patient satisfaction surveys). However, the information was seldom used to support decision making because nurse leaders did not know how to access this information. Even so, the participants agreed that the use of evidence from different sources was important in approaching any leadership or managerial problems in the organization. Participants also suggested that all nurse leaders should receive systematic training related to the topic; this could support the daily use of the evidence-based approach.

## Discussion

To our knowledge, this article represents the first mixed-methods systematic review to examine leadership problems, how evidence is used to solve these problems and what the perceived and measured effects of evidence-based leadership are on nurse leaders and their performance, organizational, and clinical outcomes. This review has two key findings. First, the available research data suggests that evidence-based leadership has potential in the healthcare context, not only to improve knowledge and skills among nurses, but also to improve organizational outcomes and the quality of patient care. Second, remarkably little published research was found to explore the effects of evidence-based leadership with an efficient trial design. We validated the preliminary results with nurse stakeholders, and confirmed that nursing staff, especially nurse leaders, were not familiar with the concept of evidence-based leadership, nor were they used to implementing evidence into their leadership decisions. Our data was based on many databases, and we screened a large number of studies. We also checked existing registers and databases and found no registered or ongoing similar reviews being conducted. Therefore, our results may not change in the near future.

We found that after identifying the leadership problems, 26 (84%) studies out of 31 used organizational data, 25 (81%) studies used scientific evidence from the literature, and 21 (68%) studies considered the views of stakeholders in attempting to understand specific leadership problems more deeply. However, only four studies critically appraised any of these findings. Considering previous critical statements of nurse leaders’ use of evidence in their decision making [14, 30, 31, 34, 55], our results are still quite promising.

Our results support a previous systematic review by Geert et al. [32], which concluded that it is possible to improve leaders’ individual-level outcomes, such as knowledge, motivation, skills, and behavior change using evidence-based approaches. Collins and Holton [23] particularly found that leadership training resulted in significant knowledge and skill improvements, although the effects varied widely across studies. In our study, evidence-based leadership was seen to enable changes in clinical practice, especially in patient care. On the other hand, we understand that not all efforts to changes were successful [56–58]. An evidence-based approach causes negative attitudes and feelings. Negative emotions in participants have also been reported due to changes, such as discomfort with a new working style [59]. Another study reported inconvenience in using a new intervention and its potential risks for patient confidentiality. Sometimes making changes is more time consuming than continuing with current practice [60]. These findings may partially explain why new interventions or program do not always fully achieve their goals. On the other hand, Dubose et al. [61] state that, if prepared with knowledge of resistance, nurse leaders could minimize the potential negative consequences and capitalize on a powerful impact of change adaptation.

We found that only six studies used a specific model or theory to understand the mechanism of change that could guide leadership practices. Participants’ reactions to new approaches may be an important factor in predicting how a new intervention will be implemented into clinical practice. Therefore, stronger effort should be put to better understanding the use of evidence, how participants’ reactions and emotions or practice changes could be predicted or supported using appropriate models or theories, and how using these models are linked with leadership outcomes. In this task, nurse leaders have an important role. At the same time, more responsibilities in developing health services have been put on the shoulders of nurse leaders who may already be suffering under pressure and increased burden at work. Working in a leadership position may also lead to role conflict. A study by Lalleman et al. [62] found that nurses were used to helping other people, often in ad hoc situations. The helping attitude of nurses combined with structured managerial role may cause dilemmas, which may lead to stress. Many nurse leaders opt to leave their positions less than 5 years [63].To better fulfill the requirements of health services in the future, the role of nurse leaders in evidence-based leadership needs to be developed further to avoid ethical and practical dilemmas in their leadership practices.

It is worth noting that the perceived and measured effects did not offer strong support to each other but rather opened a new venue to understand the evidence-based leadership. Specifically, the perceived effects did not support to measured effects (competence, ability to understand patients’ needs, use of resources, team effort, and specific clinical outcomes) while the measured effects could not support to perceived effects (nurse’s performance satisfaction, changes in practices, and clinical outcomes satisfaction). These findings may indicate that different outcomes appear if the effects of evidence-based leadership are looked at using different methodological approach. Future study is encouraged using well-designed study method including mixed-method study to examine the consistency between perceived and measured effects of evidence-based leadership in health care.

There is a potential in nursing to support change by demonstrating conceptual and operational commitment to research-based practices [64]. Nurse leaders are well positioned to influence and lead professional governance, quality improvement, service transformation, change and shared governance [65]. In this task, evidence-based leadership could be a key in solving deficiencies in the quality, safety of care [14] and inefficiencies in healthcare delivery [12, 13]. As WHO has revealed, there are about 28 million nurses worldwide, and the demand of nurses will put nurse resources into the specific spotlight [1]. Indeed, evidence could be used to find solutions for how to solve economic deficits or other problems using leadership skills. This is important as, when nurses are able to show leadership and control in their own work, they are less likely to leave their jobs [66]. On the other hand, based on our discussions with stakeholders, nurse leaders are not used to using evidence in their own work. Further, evidence-based leadership is not possible if nurse leaders do not have access to a relevant, robust body of evidence, adequate funding, resources, and organizational support, and evidence-informed decision making may only offer short-term solutions [55]. We still believe that implementing evidence-based strategies into the work of nurse leaders may create opportunities to protect this critical workforce from burnout or leaving the field [67]. However, the role of the evidence-based approach for nurse leaders in solving these problems is still a key question.

### Limitations

This study aimed to use a broad search strategy to ensure a comprehensive review but, nevertheless, limitations exist: we may have missed studies not included in the major international databases. To keep search results manageable, we did not use specific databases to systematically search grey literature although it is a rich source of evidence used in systematic reviews and meta-analysis [68]. We still included published conference abstract/proceedings, which appeared in our scientific databases. It has been stated that conference abstracts and proceedings with empirical study results make up a great part of studies cited in systematic reviews [69]. At the same time, a limited space reserved for published conference publications can lead to methodological issues reducing the validity of the review results [68]. We also found that the great number of studies were carried out in western countries, restricting the generalizability of the results outside of English language countries. The study interventions and outcomes were too different across studies to be meaningfully pooled using statistical methods. Thus, our narrative synthesis could hypothetically be biased. To increase transparency of the data and all decisions made, the data, its categorization and conclusions are based on original studies and presented in separate tables and can be found in Additional files. Regarding a methodological approach [34], we used a mixed methods systematic review, with the core intention of combining quantitative and qualitative data from primary studies. The aim was to create a breadth and depth of understanding that could confirm to or dispute evidence and ultimately answer the review question posed [34, 70]. Although the method is gaining traction due to its usefulness and practicality, guidance in combining quantitative and qualitative data in mixed methods systematic reviews is still limited at the theoretical stage [40]. As an outcome, it could be argued that other methodologies, for example, an integrative review, could have been used in our review to combine diverse methodologies [71]. We still believe that the results of this mixed method review may have an added value when compared with previous systematic reviews concerning leadership and an evidence-based approach.

## Conclusions

Our mixed methods review fills the gap regarding how nurse leaders themselves use evidence to guide their leadership role and what the measured and perceived impact of evidence-based leadership is in nursing. Although the scarcity of controlled studies on this topic is concerning, the available research data suggest that evidence-based leadership intervention can improve nurse performance, organizational outcomes, and patient outcomes. Leadership problems are also well recognized in healthcare settings. More knowledge and a deeper understanding of the role of nurse leaders, and how they can use evidence in their own managerial leadership decisions, is still needed. Despite the limited number of studies, we assume that this narrative synthesis can provide a good foundation for how to develop evidence-based leadership in the future.

### Implications

Based on our review results, several implications can be recommended. First, the future of nursing success depends on knowledgeable, capable, and strong leaders. Therefore, nurse leaders worldwide need to be educated about the best ways to manage challenging situations in healthcare contexts using an evidence-based approach in their decisions. This recommendation was also proposed by nurses and nurse leaders during our discussion meeting with stakeholders.

Second, curriculums in educational organizations and on-the-job training for nurse leaders should be updated to support general understanding how to use evidence in leadership decisions. And third, patients and family members should be more involved in the evidence-based approach. It is therefore important that nurse leaders learn how patients’ and family members’ views as stakeholders are better considered as part of the evidence-based leadership approach.

Future studies should be prioritized as follows: establishment of clear parameters for what constitutes and measures evidence-based leadership; use of theories or models in research to inform mechanisms how to effectively change the practice; conducting robust effectiveness studies using trial designs to evaluate the impact of evidence-based leadership; studying the role of patient and family members in improving the quality of clinical care; and investigating the financial impact of the use of evidence-based leadership approach within respective healthcare systems.

### Electronic supplementary material

Below is the link to the electronic supplementary material.

Supplementary Material 1

Supplementary Material 2

Supplementary Material 3

Supplementary Material 4

Supplementary Material 5

Supplementary Material 6

Supplementary Material 7

Supplementary Material 8

Supplementary Material 9

Supplementary Material 10

## Data Availability

The authors obtained all data for this review from published manuscripts.

## Declarations

### Differences between the original protocol

We modified criteria for the included studies: we included published conference abstracts/proceedings, which form a relatively broad knowledge base in scientific knowledge. We originally planned to conduct a survey with open-ended questions followed by a face-to-face meeting to discuss the preliminary results of the review. However, to avoid extra burden in nurses due to COVID-19, we decided to limit the validation process to the online discussion only.
