# Supplementary material for: The impact of evidence-based nursing leadership in healthcare settings: a mixed methods systematic review
Source: BMC Nurs. 2024 Jul 3;23:452. doi: 10.1186/s12912-024-02096-4 (PMC11221094; doi:10.1186/s12912-024-02096-4)
Supplement: Supplementary file 1 — Supplementary Material 1 [file 12912_2024_2096_MOESM1_ESM.docx]

**Additional file 1: The search strategies used in different databases and the number of hits**

| **Database** | **Search terms** | **Number of hits** |
| --- | --- | --- |
| CINAHL (EBSCO) | ("nursing leadership*" OR "leadership in nursing*" OR "nurse leader*" OR "nurse manag*" OR "nursing manag*" OR "nursing supervisor*" OR "nurse supervisor*" OR MH "Nursing Management" OR "director of nursing*" OR "nursing director*" OR "nurse director*" OR "nurse administrator*" OR MH "Nurse Administrators" OR "nursing administrator*" OR "nurse executive*" OR "executive nurse*" OR "primary nurse*" OR "chief nurse*" OR "chief nursing officer*" OR "head nurse*" OR matron* OR "charge nurse*" OR "sister nurse*" OR "ward sister*" OR "nurse executive*" OR "nursing executive*" OR "unit manag*") AND ("evidence based leadership*" OR EBL OR "evidence based management*" OR EBM OR "evidence based practice*" OR MH "Professional Practice, Evidence-Based" OR EBP OR "evidence based nursing*" OR MH "Nursing Practice, Evidence-Based" OR EBN OR "evidence based health*" OR "evidence-informed" OR "evidence informed*") AND (leadership* OR lead* OR MH "Leadership" OR manag* OR organiz* OR MH "Management" OR influenc* OR "peer influence*" OR administrat* OR superv*) | 1160 |
| Cochrane Library | (nursing NEXT leadership* OR leadership NEXT in NEXT nursing* OR nurse NEXT leader* OR nurse NEXT manag* OR nursing NEXT manag* OR nursing NEXT supervisor* OR nurse NEXT supervisor* OR director NEXT of NEXT nursing* OR nursing NEXT director* OR nurse NEXT director* OR nurse NEXT administrator* OR nursing NEXT administrator* OR nurse NEXT executive* OR executive NEXT nurse* OR primary NEXT nurse* OR chief NEXT nurse* OR chief NEXT nursing officer* OR head NEXT nurse* OR matron* OR charge NEXT nurse* OR sister NEXT nurse* OR ward NEXT sister* OR nurse NEXT executive* OR nursing NEXT executive* OR unit NEXT manag*) AND (evidence NEXT based NEXT leadership* OR EBL OR evidence NEXT based NEXT management* OR EB OR evidence NEXT based NEXT practice* OR EBP OR evidence NEXT based NEXT nursing* OR EBN OR evidence NEXT based NEXT health* OR evidence-informed* OR evidence NEXT informed*) AND (leadership* OR lead* OR manag* OR organiz* OR influenc* OR administrat* OR superv*) | 15 |
| Embase (Elsevier) | ("nursing leadership*" OR "leadership in nursing*" OR "nurse leader*" OR "nurse manag*" OR "nursing manag*" OR "nursing supervisor*" OR "nurse supervisor*" OR 'nursing management'/exp OR "director of nursing*" OR "nursing director*" OR "nurse director*" OR "nurse administrator*" OR 'nurse administrator'/exp OR "nursing administrator*" OR "nurse executive*" OR "executive nurse*" OR "primary nurse*" OR "chief nurse*" OR "chief nursing officer*" OR "head nurse*" OR matron* OR "charge nurse*" OR "sister nurse*" OR "ward sister*" OR "nurse executive*" OR "nursing executive*" OR "unit manag*") AND ("evidence based leadership*" OR EBL OR "evidence based management*" OR EBM OR "evidence based practice*" OR 'evidence based practice'/exp OR EBP OR "evidence based nursing*" OR 'evidence based nursing'/exp OR EBN OR "evidence based health*" OR "evidence-informed" OR "evidence informed*") AND (leadership* OR lead* OR 'leadership'/exp OR manag* OR organiz* OR 'management'/exp OR influenc* OR "peer influence*" OR administrat* OR superv*) | 2264 |
| PsycINFO (EBSCO) | ("nursing leadership*" OR "leadership in nursing*" OR "nurse leader*" OR "nurse manag*" OR "nursing manag*" OR "nursing supervisor*" OR "nurse supervisor*" OR "director of nursing*" OR "nursing director*" OR "nurse director*" OR "nurse administrator*" OR "nursing administrator*" OR "nurse executive*" OR "executive nurse*" OR "primary nurse*" OR "chief nurse*" OR "chief nursing officer*" OR "head nurse*" OR matron* OR "charge nurse*" OR "sister nurse*" OR "ward sister*" OR "nurse executive*" OR "nursing executive*" OR "unit manag*") AND ("evidence based leadership*" OR EBL OR "evidence based management*" OR EBM OR "evidence based practice*" OR DE "evidence based practice" OR EBP OR "evidence based nursing*" OR EBN OR "evidence based health*" OR "evidence-informed" OR "evidence informed*") AND (leadership* OR lead* OR DE "Leadership" OR manag* OR organiz* OR DE "Management" OR influenc* OR "peer influence*" OR administrat* OR superv*) | 301 |
| PubMed  (MEDLINE) | ("nursing leadership*"[tw] OR "leadership in nursing*"[tw] OR "nurse leader*"[tw] OR "nurse manag*"[tw] OR "nursing manag*"[tw] OR "nursing supervisor*"[tw] OR "nurse supervisor*"[tw] OR "Nursing, Supervisory"[Mesh] OR "director of nursing*"[tw] OR "nursing director*"[tw] OR "nurse director*"[tw] OR "nurse administrator*"[tw] OR "Nurse Administrators"[Mesh] OR "nursing administrator*"[tw] OR "nurse executive*"[tw] OR "executive nurse*"[tw] OR "primary nurse*"[tw] OR "chief nurse*"[tw] OR "chief nursing officer*"[tw] OR "head nurse*"[tw] OR matron*[tw] OR "charge nurse*"[tw] OR "sister nurse*"[tw] OR "ward sister*"[tw] OR "nurse executive*"[tw] OR "nursing executive*"[tw] OR "unit manag*"[tw]) AND ("evidence based leadership*"[tw] OR EBL[tw] OR "evidence based management*"[tw] OR EBM[tw] OR "evidence based practice*"[tw] OR "Evidence-Based Practice"[Mesh] OR EBP[tw] OR "evidence based nursing*"[tw] OR "Evidence-Based nursing"[Mesh] OR EBN[tw] OR "evidence based health*"[tw] OR evidence-informed[tw] OR "evidence informed*"[tw]) AND (leadership*[tw] OR lead*[tw] OR "Leadership"[Mesh] OR manag*[tw] OR organiz*[tw] OR "Organization and Administration"[Mesh] OR influenc*[tw] OR "Peer Influence"[Mesh] OR administrat*[tw] OR superv*[tw]) | 932 |
| Scopus (Elsevier) | ("nursing leadership*" OR "leadership in nursing*" OR "nurse leader*" OR "nurse manag*" OR "nursing manag*" OR "nursing supervisor*" OR "nurse supervisor*" OR "director of nursing*" OR "nursing director*" OR "nurse director*" OR "nurse administrator*" OR "nursing administrator*" OR "nurse executive*" OR "executive nurse*" OR "primary nurse*" OR "chief nurse*" OR "chief nursing officer*" OR "head nurse*" OR matron* OR "charge nurse*" OR "sister nurse*" OR "ward sister*" OR "nurse executive*" OR "nursing executive*" OR "unit manag*") AND ("evidence based leadership*" OR EBL OR "evidence based management*" OR EBM OR "evidence based practice*" OR EBP OR "evidence based nursing*" OR EBN OR "evidence based health*" OR "evidence-informed" OR "evidence informed*") AND (leadership* OR lead* OR manag* OR organiz* OR influenc* OR "peer influence*" OR administrat* OR superv*) | 990 |
| Web of Science | (TS=(("nursing leadership*" OR "leadership in nursing*" OR "nurse leader*" OR "nurse manag*" OR "nursing manag*" OR "nursing supervisor*" OR "nurse supervisor*" OR "director of nursing*" OR "nursing director*" OR "nurse director*" OR "nurse administrator*" OR "nursing administrator*" OR "nurse executive*" OR "executive nurse*" OR "primary nurse*" OR "chief nurse*" OR "chief nursing officer*" OR "head nurse*" OR matron* OR "charge nurse*" OR "sister nurse*" OR "ward sister*" OR "nurse executive*" OR "nursing executive*" OR "unit manag*") AND ("evidence based leadership*" OR EBL OR "evidence based management*" OR EBM OR "evidence based practice*" OR EBP OR "evidence based nursing*" OR EBN OR "evidence based health*" OR "evidence-informed" OR "evidence informed*") AND (leadership* OR lead* OR manag* OR organiz* OR influenc* OR "peer influence*" OR administrat* OR superv*)) | 391 |
