# Supplementary material for: The impact of evidence-based nursing leadership in healthcare settings: a mixed methods systematic review
Source: BMC Nurs. 2024 Jul 3;23:452. doi: 10.1186/s12912-024-02096-4 (PMC11221094; doi:10.1186/s12912-024-02096-4)
Supplement: Supplementary file 2 — Supplementary Material 2 [file 12912_2024_2096_MOESM2_ESM.docx]

**Additional file 2: The full references list of included studies**

Ref 1. Alleyne J, Jumaa MO. Building the capacity for evidence-based clinical nursing leadership: the role of executive co-coaching and group clinical supervision for quality patient services. Journal of Nursing Management. 2007;15(2): 230–243. https://doi.org/10.1111/j.1365-2834.2007.00750.x.

Ref 2. Busbee V, Lindsay S, Mayernik C, Ramey J. System Culture Change Decreases CAUTI. American Journal of Infection Control. 2020;48: S15−S58.

Ref 3. Cullen L, Titler MG. Promoting Evidence-Based Practice: An Internship for Staff Nurses. Worldviews on Evidence-Based Nursing. 2004;1(4): 215–223. https://doi.org/10.1111/j.1524-475X.2004.04027.x.

Ref 4. Davidson JE, Brown C. Evaluation of Nurse Engagement in Evidence-Based Practice. AACN Advanced Critical Care. 2014;25(1): 43–55. https://doi.org/10.4037/NCI.0000000000000006.

Ref 5. DeLeskey K. The implementation of evidence-based practice for the prevention/management of post-operative nausea and vomiting. International Journal of Evidence-Based Healthcare. 2009;7(2): 140–144. https://doi.org/10.1111/j.1744-1609.2009.00131.x.

Ref 6. Galiano A, Simonetti M, Quiroga N, Larrain A. Development, implementation and evaluation of an evidence‐based practice model in a new hospital in Chile. Journal of Nursing Management. 2020;28(7): 1748–1757. https://doi.org/10.1111/jonm.13134.

Ref 7. Gifford W, Davies B, Tourangeau A, Lefebre N. Developing team leadership to facilitate guideline utilization: planning and evaluating a 3-month intervention strategy: Team leadership to facilitate guideline utilization. Journal of Nursing Management. 2011;19(1): 121–132. https://doi.org/10.1111/j.1365-2834.2010.01140.x.

Ref 8. Gifford W, Davies BL, Graham ID, Tourangeau A, Woodend AK, Lefebre N. Developing Leadership Capacity for Guideline Use: A Pilot Cluster Randomized Control Trial: Leadership Pilot Study. Worldviews on Evidence-Based Nursing. 2013;10(1): 51–65. https://doi.org/10.1111/j.1741-6787.2012.00254.x.

Ref 9. Gifford W, Lefebre N, Davies B. An Organizational Intervention to Influence Evidence-Informed Decision Making in Home Health Nursing. JONA: The Journal of Nursing Administration. 2014;44(7/8): 395–402. https://doi.org/10.1097/NNA.0000000000000089.

Ref 10. Hester JM, Perrin KK, Reed RR, Larson AA. Mission Impossible: Achieving Zero CAUTIs in the NeuroIntensive Care Unit Using a Patient Centered, Interdisciplinary Team Approach. Neurocrit Care. 2016;(25): S1–S310.

Ref 11. Hoke N, Bradway C. A Clinical Nurse Specialist–Directed Initiative to Reduce Postoperative Urinary Retention in Spinal Surgery Patients. AJN The American Journal of Nursing. 2016;116(8): 47–52. https://doi.org/10.1097/01.NAJ.0000490176.22393.69.

Ref 12. Hsieh HY, Henker R, Ren D, Chien WY, Chang JP, Chen L, et al. Improving Effectiveness and Satisfaction of an Electronic Charting System in Taiwan. Clinical Nurse Specialist. 2016;30(6): E1–E6. https://doi.org/10.1097/NUR.0000000000000250.

Ref 13. Kidd S, Christiansen K, Coumar A, Williams J, Ito K, Petersen A, et al. A Dedicated Education Unit and a Novel Resident Nurse Transition-to-Practice Program in an Ambulatory Oncology Setting. Seminars in Oncology Nursing. 2020;36(3): 151027. https://doi.org/10.1016/j.soncn.2020.151027.

Ref 14. Kneflin N, O’Quinn L, Geigle G, Mott B, Nebrig D, Munafo J. Direct care nurses on the shared governance journey towards positive patient outcomes. Journal of Clinical Nursing. 2016;25(5–6): 875–882. https://doi.org/10.1111/jocn.13114.

Ref 15. Laws D, Crawford CL. Alternative Strategies to Constant Patient Observation and Sitters: A Proactive Approach. JONA: The Journal of Nursing Administration. 2013;43(10): 497–501. https://doi.org/10.1097/NNA.0b013e3182a3e83e.

Ref 16. McAllen E, Stephens K, Swanson-Biearman B, Kerr K, Whiteman K. Moving Shift Report to the Bedside: An Evidence-Based Quality Improvement Project. OJIN: The Online Journal of Issues in Nursing. 2018;23(2). https://doi.org/10.3912/OJIN.Vol23No02PPT22.

Ref 17. McDonough KS, Pemberton M. Evaluation and Development of an ED Management Model: An Effort to Optimize Patient-Centered Care. Journal of Emergency Nursing. 2013;39(5): 485–490. https://doi.org/10.1016/j.jen.2013.05.004.

Ref 18. McFarlan S, O’Brien D, Simmons E. Nurse-Leader Collaborative Improvement Project: Improving Patient Experience in the Emergency Department. Journal of Emergency Nursing. 2019;45(2): 137–143. https://doi.org/10.1016/j.jen.2018.11.007.

Ref 19. McKinley C, Fletcher A, Biggins A, McMurray A, Birtwhistle S, Gardiner L, et al. Evidence-based Management Practice: Reducing Falls in Hospital. Collegian. 2007;14(2): 20–25. https://doi.org/10.1016/S1322-7696(08)60551-X.

Ref 20. Ostaszkiewicz O, Cecil J, Kosowicz L. A best practice model of continence care in residential aged care. The International Continence Society. 2021; S121.

Ref 21. Parchment J, Stinson A. Clinical Nurses: Leading Through the Complexity of Human Trafficking. Nursing Administration Quarterly. 2020;44(3): 235–243. https://doi.org/10.1097/NAQ.0000000000000423.

Ref 22. Britt Pipe T. Optimizing Nursing Care by Integrating Theory-Driven Evidence-Based Practice. Journal of Nursing Care Quality. 2007;22(3): 234–238. https://doi.org/10.1097/01.NCQ.0000277780.27771.91.

Ref 23. Robbins JR, Valdez-Delgado KK, Caldwell NW, Yoder LH, Hayes EJ, Barba MG, et al. Implementation and outcomes of an evidence-based precepting program for burn nurses. Burns. 2017;43(7): 1441–1448. https://doi.org/10.1016/j.burns.2017.04.017.

Ref 24. Salvador P, Howell D. Development of a symptom distress reduction model for stem cell transplant patients. Psycho-Oncology. 2010;19(Suppl. 2): S1–S313.

Ref 25. Stacey D, Ludwig C, Truant T, Carley M, Bennis C, Gifford W, et al. Implementing Practice Guides to Improve Cancer Symptom Management in Homecare: A Comparative Case Study. Home Health Care Management & Practice. 2019;31(3): 139–146. https://doi.org/10.1177/1084822318817896.

Ref 26. Sving E, Fredriksson L, Mamhidir AG, Högman M, Gunningberg L. A multifaceted intervention for evidence-based pressure ulcer prevention: a 3 year follow-up. International Journal of Evidence-Based Healthcare. 2020;Publish Ahead of Print. https://doi.org/10.1097/XEB.0000000000000239.

Ref 27. Tafelmeyer J, Wicks R, Brant J, Smith L. Incorporating Nurse Input and Evidence Into a Newly Designed Unit to Improve Patient and Nursing Outcomes. JONA: The Journal of Nursing Administration. 2017;47(12): 603–609. https://doi.org/10.1097/NNA.0000000000000554.

Ref 28. Thomas, Donohue-Porter P. Blending Evidence and Innovation: Improving Intershift Handoffs in a Multihospital Setting. Journal of Nursing Care Quality. 2012;27(2): 116–124. https://doi.org/10.1097/NCQ.0b013e318241cb3b.

Ref 29. Thomas M, Autencio K, Cesario K. Positive outcomes of an evidence-based pressure injury prevention program. Journal of Wound Ostomy & Continence Nursing. 2020;47: S24.

Ref 30. Van Diggele C, Burgess A, Roberts C, Mellis C. Leadership in healthcare education. BMC Medical Education. 2020;20(2): 1–6.

Ref 31. Yurumezoglu HA, Kocaman G. Pilot study for evidence-based nursing management: Improving the levels of job satisfaction, organizational commitment, and intent to leave among nurses in Turkey: Evidence-based nursing management. Nursing & Health Sciences. 2012;14(2): 221–228. https://doi.org/10.1111/j.1442-2018.2012.00682.x.
