# Supplementary material for: The impact of evidence-based nursing leadership in healthcare settings: a mixed methods systematic review
Source: BMC Nurs. 2024 Jul 3;23:452. doi: 10.1186/s12912-024-02096-4 (PMC11221094; doi:10.1186/s12912-024-02096-4)
Supplement: Supplementary file 3 — Supplementary Material 3 [file 12912_2024_2096_MOESM3_ESM.docx]

**Additional file 3: Narrative description of the study characteristics**

***Type of studies***

The studies were of a wide variety of types. The clear majority were projects aiming to develop the current healthcare system. The studies were practice development projects (ref 2, ref 13, ref 18, ref 22), action research (ref 1), and implementation projects (ref 4, ref 5, ref 6, ref 7, ref 9, ref 10, ref 11, ref 12, ref 15, ref 17, ref 23, ref 28, ref 29). Quality improvement projects (ref 16, ref 21, ref 30) and quality management initiative projects were also examined (ref 19). A multimethod approach (ref 3, ref 20), a sequential mixed-methods approach alone (ref 8, ref 26), a sequential mixed-methods approach with a post-only cluster randomized clinical trial (ref 8), and a single-group, quasi-experimental, pro-post-test with repeated measures were used (ref 31). In addition, a pre-post assessment was conducted (ref 27). Other study types were used, such as positioning paper (ref 14), a model development project (ref 24) and a comparative case study (see Table 2 for description of the characteristics of included studies) (ref 25).

***Country where the study was conducted***

Most studies were conducted in western countries. The clear majority were conducted in the USA (n=19) (61%) (ref 2, ref 3, ref 4, ref 5, ref 10, ref 11, ref 13, ref 14, ref 15, ref 16, ref 17, ref 18, ref 21, ref 22, ref 23, ref 27, ref 28, ref 29, ref 30). Other studies were conducted in Canada (n=5) (16%) (ref 7, ref 8, ref 9, ref 24, ref 25), and Australia (n=2) (6%) (ref 19, ref 20), the UK (n=1) (3%) (ref 1), Turkey (n=1) (3%) (ref 31), Chile (n=1) (3%) (ref 6), China (Taiwan) (n=1) (3%) (ref 12) and Sweden (n=1) (3%) (see Table 2 for description of the characteristics of included studies) (ref 26).

***Participants***

Typically, the study participants were staff nurses (n=14) (45%) (ref 4, ref 6, ref 10, ref 12, ref 13, ref 16, ref 20, ref 22, ref 23, ref 24, ref 25, ref 27, ref 28, ref 30). Nurses in managerial positions included nurse managers, clinical leaders or facility leads (n=5) (16%) (ref 2, ref 7, ref 8, ref 9, ref 15). A combination of district nurses and doctoral candidates (n=1) (3%) (ref 1) and nurses and nurse managers (n=2) (6%) (ref 3, ref 14) was included in the studies. Studies on nurses (ref 31) and patients alone (n=2) (6%) (ref 5, ref 26) were also reported. In some cases, the whole population of a hospital (n=2) (6%) (ref 19, ref 29) or department staff (n=1) (3%) (ref 17) were participants in the study. Multiple professional groups (n=2) (6%) were also included (ref 18, ref 21). One study used patient records as an information source (3%) (see Table 2 for description of the characteristics of included studies) (ref 11).

Nineteen out of 31 studies (61%) reported a specific number of participants in the study. The size of the participant groups varied between 8 (ref 1) and 510 (ref 6). Most studies were conducted in hospital settings (ref 1, ref 2, ref 3, ref 4, ref 5, ref 6, ref 10, ref 11, ref 12, ref 13, ref 14, ref 15, ref 16, ref 17, ref 18, ref 19, ref 22, ref 23, ref 24, ref 26, ref 27, ref 28, ref 29, ref 30, ref 31). Other settings were home and community healthcare organizations (ref 7), homecare organizations (ref 8, ref 9), nursing agencies (ref 25) and aged care homes (ref 20). The study setting of one study was not mentioned (see Table 2 for description of the characteristics of included studies) (ref 21).
