# Supplementary material for: The impact of evidence-based nursing leadership in healthcare settings: a mixed methods systematic review
Source: BMC Nurs. 2024 Jul 3;23:452. doi: 10.1186/s12912-024-02096-4 (PMC11221094; doi:10.1186/s12912-024-02096-4)
Supplement: Supplementary file 4 — Supplementary Material 4 [file 12912_2024_2096_MOESM4_ESM.docx]

**Additional file 4: Critical appraisal of included studies**

**Critical appraisal of a quasi-experimental study**

| **Author(s) (year)**  **(Ref #)** | **Q1** | **Q2** | **Q3** | **Q4** | **Q5** | **Q6** | **Q7** | **Q8** | **Q9** | **Score** |
| --- | --- | --- | --- | --- | --- | --- | --- | --- | --- | --- |
| Yurumezoglu & Kocaman (2012)  (Ref 31) | Y | Y | Y | N | Y | Y | Y | Y | Y | 8/9 |

Y, yes; N, no; U, unclear; NA, not applicable; JBI critical appraisal checklist for quasi-experimental studies.

Q1: Is it clear in the study what is the ‘‘cause’’ and what is the ‘‘effect’’ (ie, there is no confusion about which variable comes first)?

Q2: Were the participants included in any comparisons similar?

Q3: Were the participants included in any comparisons receiving similar treatment/care, other than the exposure or intervention of interest?

Q4: Was there a control group?

Q5: Were there multiple measurements of the outcome both pre and post the intervention/exposure?

Q6: Was follow-up complete and if not, were differences between groups in terms of their follow-up adequately described and analyzed?

Q7: Were the outcomes of participants included in any comparisons measured in the same way?

Q8: Were outcomes measured in a reliable way?

Q9: Was appropriate statistical analysis used?

**Critical appraisal of case series studies**

| **Author(s) (year)**  **(Ref #)** | **Q1** | **Q2** | **Q3** | **Q4** | **Q5** | **Q6** | **Q7** | **Q8** | **Q9** | **Q10** | **Score** |
| --- | --- | --- | --- | --- | --- | --- | --- | --- | --- | --- | --- |
| Alleyne & Jumaa (2007)  (Ref 1) | Y | U | NA | U | U | N | Y | Y | N | U | 3/9 |
| Busbee et al. (2020 a,b)  (Ref 2) | U | U | U | U | U | U | U | Y | U | U | 1/10* |
| Cullen & Titler (2004)  (Ref 3) | Y | Y | NA | U | U | N | Y | Y | N | U | 4/9 |
| Davidson & Brown (2014)  (Ref 4) | N | Y | NA | Y | Y | N | Y | Y | N | U | 5/9 |
| DeLeskey (2009)  (Ref 5) | Y | Y | Y | Y | Y | N | Y | Y | N | Y | 8/10 |
| Galiano et al. (2020)  (Ref 6) | N | N | NA | U | U | N | Y | Y | N | Y | 3/9 |
| Gifford et al. (2011)  (Ref 7) | Y | U | NA | U | Y | Y | Y | Y | N | Y | 6/9 |
| Hester et al. (2016)  (Ref 10) | U | U | U | U | U | U | U | Y | U | Y | 2/10* |
| Hoke et al. (2016)  (Ref 11) | N | Y | Y | Y | U | N | N | Y | Y | Y | 6/10 |
| Hsieh et al. (2016)  (Ref 12) | N | Y | NA | Y | Y | N | Y | Y | Y | Y | 7/9 |
| Kidd et al. (2020)  (Ref 13) | Y | U | NA | U | Y | N | Y | Y | Y | Y | 6/9 |
| Kneflin et al. (2016)  (Ref 14) | Y | U | NA | U | Y | N | Y | Y | Y | U | 5/9 |
| Laws et al. (2013)  (Ref 15) | N | U | NA | Y | U | N | Y | Y | Y | U | 4/9 |
| McAllen et al. (2018)  (Ref 16) | Y | U | N | Y | Y | N | Y | Y | Y | Y | 7/10 |
| McDonough & Pemberton (2013)  (Ref 17) | Y | Y | NA | Y | Y | N | Y | Y | Y | U | 7/9 |
| McFarlan et al. (2019)  (Ref 18) | Y | N | NA | Y | U | N | Y | Y | Y | U | 5/9 |
| McKinley et al.(2007)  (Ref 19) | N | N | Y | Y | Y | N | N | Y | N | Y | 5/10 |
| Ostaszkiewicz et al. (2021)  (Ref 20) | U | U | U | U | U | U | U | Y | U | U | 1/10* |
| Parchment & Stinson (2020)  (Ref 21) | U | N | NA | Y | N | N | Y | N | Y | U | 3/9 |
| Britt Pipe (2007)  (Ref 22) | N | Y | N | U | U | N | N | N | Y | U | 2/10 |
| Robbins et al. (2017)  (Ref 23) | Y | Y | Y | Y | U | N | Y | Y | Y | U | 7/10 |
| Salvador & Howell (2010)  (Ref 24) | U | U | Y | U | U | U | U | U | U | U | 1/10* |
| Stacey et al. (2019)  (Ref 25) | Y | Y | Y | Y | Y | Y | N | Y | Y | Y | 9/10 |
| Tafelmeyer (2017)  (Ref 27) | N | U | NA | U | U | N | N | Y | N | U | 1/9 |
| Thomas & Donohue-Porter (2012)  (Ref 28) | Y | Y | U | Y | Y | N | Y | Y | Y | U | 7/10 |
| Thomas et al. (2020)  (Ref 29) | U | U | Y | Y | U | U | U | Y | U | U | 3/10* |
| Van Orne (2021)  (Ref 30) | Y | Y | Y | Y | Y | N | Y | Y | N | Y | 8/10 |

*Only abstract available

Y, yes; N, no; U, unclear; NA, not applicable; JBI critical appraisal checklist for case series.

Q1: Were there clear criteria for inclusion in the case series?

Q2: Was the condition measured in a standard, reliable way for all participants included in the case series?

Q3: Were valid methods used for identification of the condition for all participants included in the case series?

Q4: Did the case series have consecutive inclusion of participants?

Q5: Did the case series have complete inclusion of participants?

Q6: Was there clear reporting of the demographics of the participants in the study?

Q7: Was there clear reporting of clinical information of the participants?

Q8: Were the outcomes or follow up results of cases clearly reported?

Q9: Was there clear reporting of the presenting site(s)/clinic(s) demographic information?

Q10: Was statistical analysis appropriate?

**Critical appraisal for mixed methods studies**

| **Author(s) (year)**  **(Ref #)** | S1 | S1 | 1.1 | 1.2 | 1.3 | 1.4 | 1.5 | 2.1 | 2.2 | 2.3 | 2.4 | 2.5 | 3.1 | 3.2 | 3.3 | 3.4 | 3.5 | 4.1 | 4.2 | 4.3 | 4.4 | 4.5 | 5.1 | 5.2 | 5.3 | 5.4 | 5.5 | Score |
| --- | --- | --- | --- | --- | --- | --- | --- | --- | --- | --- | --- | --- | --- | --- | --- | --- | --- | --- | --- | --- | --- | --- | --- | --- | --- | --- | --- | --- |
| Gifford et al. (2013)  (Ref 8) | Y | Y | Y | Y | Y | Y | Y | Y | Y | Y | Y | Y |  |  |  |  |  |  |  |  |  |  | Y | Y | Y | Y | Y | 100% |
| Gifford et al. (2014)  (Ref 9) | Y | Y | Y | Y | Y | Y | Y |  |  |  |  |  |  |  |  |  |  | C | Y | Y | C | Y | Y | Y | Y | Y | N | 60% |
| Sving et al. (2020)  (Ref 26) | Y | Y | Y | Y | Y | Y | Y |  |  |  |  |  |  |  |  |  |  | Y | Y | Y | Y | Y | Y | Y | Y | Y | Y | 100% |

Y, yes; N, no; C, Can’t tell; MMAT checklist. Critical appraisal checklist for mixed methods studies.

S1. Are there clear research questions?

S2: Do the collected data allow to address the research questions?

1.1: Is the qualitative approach appropriate to answer the research question?

1.2: Are the qualitative data collection methods adequate to address the research question?

1.3. Are the findings adequately derived from the data?

1.4. Is the interpretation of results sufficiently substantiated by data?

1.5. Is there coherence between qualitative data sources, collection, analysis and interpretation?

2.1. Is randomization appropriately performed?

2.2. Are the groups comparable at baseline?

2.3. Are there complete outcome data?

2.4. Are outcome assessors blinded to the intervention provided?

2.5 Did the participants adhere to the assigned intervention?

3.1. Are the participants representative of the target population?

3.2. Are measurements appropriate regarding both the outcome and intervention (or exposure)?

3.3. Are there complete outcome data?

3.4. Are the confounders accounted for in the design and analysis?

3.5. During the study period, is the intervention administered (or exposure occurred) as intended?

4.1. Is the sampling strategy relevant to address the research question?

4.2. Is the sample representative of the target population?

4.3. Are the measurements appropriate?

4.4. Is the risk of nonresponse bias low?

4.5. Is the statistical analysis appropriate to answer the research question?

5.1. Is there an adequate rationale for using a mixed methods design to address the research question?

5.2. Are the different components of the study effectively integrated to answer the research question?

5.3. Are the outputs of the integration of qualitative and quantitative components adequately interpreted?

5.4. Are divergences and inconsistencies between quantitative and qualitative results adequately addressed?

5.5. Do the different components of the study adhere to the quality criteria of each tradition of the methods involved?
