# Supplementary material for: The impact of evidence-based nursing leadership in healthcare settings: a mixed methods systematic review
Source: BMC Nurs. 2024 Jul 3;23:452. doi: 10.1186/s12912-024-02096-4 (PMC11221094; doi:10.1186/s12912-024-02096-4)
Supplement: Supplementary file 5 — Supplementary Material 5 [file 12912_2024_2096_MOESM5_ESM.docx]

**Additional file 5: Description of interventions based on** **TiDier [43] checklist**

| **Author(s)**  **(year)**  **(Ref #)** | **Name**  **The goal of the intervention** | **Theory or background** | **Material and**  **procedure** | **Providers** | **Models of delivery and location** | **Dose** | **Modifi-cation** | **Adherence**  **and fidelity** |
| --- | --- | --- | --- | --- | --- | --- | --- | --- |
| Alleyne & Jumaa (2007)  (Ref 1) | Group Clinical Supervision (GCS); an executive co-coaching.  To offer new ways of clinical supervision to improve the quality of patient management and services. | Clinical Nursing Leadership Learning and Action Process (CLINLAP) Framework | Stakeholder Mapping and Management; various techniques, tools, methods, and frameworks. | PhD student and an accredited facilitator | Collaborative enquiry in sessions  Hospital | Group Clinical Supervision (GCS) sessions, 90 minutes weekly. | Novel method | NA |
| Busbee et al. (2020 a,b)  (Ref 2) | No name.  To improve the standardization of products, insertion techniques, and the development of nurse driven standing orders for removal were needed to eradicate system wide CAUTI. | NA | System-wide culture change and standardization. Developing a multidisciplinary team, following in-depth review of CAUTI cases and analysis of the data, several evidence-based practice CAUTI reduction strategies were implemented. | Nurse | NA  Hospital | NA | No | NA |
| Cullen & Titler (2004)  (Ref 3) | Internship program.  To promote use of evidence to improve patient outcome; to promote innovative thinking by nurses, to facilitate development and integration of a clinically relevant evidence-based practice project; to expand understanding and use of the Iowa model of evidence-based practice to promote quality care; to promote professional growth and development of staff nurse. | NA | Support from nurse manager and advanced practice nurse mentor.  Meeting days with class courses, coursework, conference and workdays. Extra support offered. | Evidence-based practice facilitator, nurse manager and advanced practice nurse mentor. | Internship meetings and quarterly meetings to support project work face to face.  Hospital | 18-24 months, 12 meeting days including three classroom days, one day attendance at conference, and eight workdays. | After program revisions. | NA |
| Davidson & Brown (2014)  (Ref 4) | “Digging for Dinosaurs”  To explore nurses’ willingness to question practice, use evidence in practice, address clinical problems through problem resolution, and overcome obstacles. Patterns of practice improvement opportunities were evaluated. | NA | Project Steps Checklist.  Nurses submit ideas, support given by face-to-face meeting about evidence-base practice. | Clinical nurses | Face to face, online  Hospital | NA | No | NA |
| DeLeskey (2009)  (Ref 5) | No name.  To assess the current use of evidence-based care in the treatment/management of post-operative nausea and vomiting (PONV), to ensure the use of evidence in the treatment/ management of PONV, to improve patient outcomes related to PONV | NA | The American Society and Perianesthesia Nurses (ASPAN) and the American Society of Anesthesiology (ASA) developed recommendations  for best practice in preventing PONV. A team of key stakeholders was created and helped to bring change. | Project leader and stakeholders | Team discussion  Hospital | NA | No | NA |
| Galiano et al. (2020)  (Ref 6) | No name.  To motivate and guide nurse  managers from other institutions who are seeking to improve the patient safety and the quality of care through EBP. | Donabedian's Quality Model | Frameworks, literature review, internal and external analyses, multi-stage implementation plan, process and outcome evaluation | Nurse coordinator of EBP, EBP mentors | Face to face  Hospital | 45-hour course, monthly meetings. | No | NA |
| Gifford et al. (2011)  (Ref 7) | No name.  To influence nurses’ use of guideline recommendations when caring for patients with diabetic foot ulcers in  home care nursing. | NA | A guideline implementation strategy. Intervention workshop, teleconferences. | Managers and clinical leaders | Face to face, teleconference  Home and community care oraganisations | One full-day interactive workshop (6 hours) and  three follow-up teleconferences (10-30 minutes). | One center, 2 day workshop instead of 1 day | NA |
| Gifford et al. (2013)  (Ref 8) | No name.  To facilitate utilization of a clinical practice guideline for assessing and managing foot ulcers for people with diabetes in community nursing practice. | NA | Interviews, chart audit, workshop, teleconferences. | Managers, supervisors, clinical resource nurses, specialized wound care nurses | Face to face, teleconference  Home healthcare organisation | 1 (2) day workshop (6 hours) three teleconferences, totally 12 weeks | One center had 2 days workshop instead of 1 day | NA |
| Gifford et al. (2014)  (Ref 9) | Multifaceted intervention  To promote evidence-informed decision making (EIDM) by nurse managers and clinical leaders in home healthcare. | NA | Workshop, support from ‘evidence facilitators’, library services, information-sharing, encouragement, and recognition activities. | Clinical and management leaders | Face to face  Home healthcare organisation | 20 weeks organization-focused intervention to field test the intervention, one 5-hour interactive educational workshop | No | NA |
| Hester et al. (2016)  (Ref 10) | Neuro ICU Interdisciplinary Comprehensive Unit Safety Program (CUSP).  To improve the urinary catheter management. | NA | Extensive literature review, adaptation to the target context. | Staff nurse driven protocol, nursing leadership and neurocritical care interdisciplinary team | Face to face in neuro ICU  Hospital | NA | No | NA |
| Hoke et al. (2016)  (Ref 11) | Quality improvement (QI).  To increase postanestesia caring unit (PACU) nursing staff’s awareness of the risk of postoperative urinary retention (POUR) and to develop updated nursing practice guidelines for the evaluation and management of POUR in a population of adult spinal surgery patients. | NA | Form a team, review current practices, develop a PACU Algorithm for Assessment of Bladder Volume, delivery of the intervention. | Post anesthesia care unit clinical nurse specialist, neuroscience clinical nurse specialist, nurse manager, nurse practitioner, physician. | Face to face  postanestesia caring unit (PACU)  Hospital | NA | No | NA |
| Hsieh et al. (2016)  (Ref 12) | No name.  To develop, implement, and evaluate the new focus templates aimed at increasing staff satisfaction and reducing documentation time. | NA | Identify the focuses used by subject unit, identify the focuses for clinical pathways, development of the focuses, implementation. | A clinical nurse specialist, senior nurse | Face to face  Hospital | NA | No | NA |
| Kidd et al. (2020)  (Ref 13) | A dedicated education unit (DEU)  To rotate student nurse and transition-to-practice program. | NA | Interdisciplinary rounds, lectures, implementation committees, certification, conference attendance, promotional opportunities, expansion of clinical practice, mentoring, preceptor workshop. | Preceptor, DEU program staff, clinical site manager, associate director | Face to face  Hospital | Preparatory orientation, 4-week weekly reflection, 12-16 week planned rotation with communication with preceptor, written and verbal assessment | No | NA |
| Kneflin et al. (2016)  (Ref 14) | Nursing-shared governance.  To make a hospital-wide practice change that positively impacted patient outcomes and strengthened the shared governance process. | NA | Problem identification, form a team, systematic evidence search, stakeholders’ discussion, form a standardized best practice. | Nurse, Nursing Professional Practice Council, Nursing professional education council | Face to face  Hospital | NA | No | NA |
| Laws et al. (2013)  (Ref 15) | CO Strategies  To identify patients at risk for behavioral issues, developed evidence-based interventions to reduce the use of sitters and constant observation, and maintained or improved patient safety | NA | Build a multi-disciplinary team, identify best practices, implementation. | Registered nurses, patient care coordinator, unit nursing management | Face to face  Hospital | NA | No | NA |
| McAllen et al. (2018)  (Ref 16) | No name.  To improve patient safety, patient satisfaction, and nurse satisfaction. | NA | Literature Review  gap analysis, implementation, scripted report, education | Nurse, nursing  administrators,  directors, staff  nurses, and a  patient | Face to face,  three nursing units  Hospital | NA | No | NA |
| McDonough & Pemberton (2013)  (Ref 17) | ED Leadership Model  To engage employees in the effort to provide patient centered, quality care. | NA | Do survey to support that the changes are needed. Development of ED Leadership Model; leadership involvement, engaging staff, communication, “Living the Mission” -manual. | Patient care managers | Face to face,  Hospital emergency department | One-week period of instruction for patient care managers, one week mapping the future for change | No | NA |
| McFarlan et al. (2019)  (Ref 18) | No name.  To increase the Hospital Consumer Assessment of Healthcare Providers and Services (HCAHPS) scores of emergency department patients, focusing on the following 5 survey questions: (1) Response to concerns/ complaints during your stay; (2) degree to which hospital staff worked as a team; (3) staff identified themselves to patients; (4) overall rating of institution; (5) likelihood to recommend. | NA | Stakeholders meeting, leadership stakeholder involvement, baseline data review, literature review, created standard workflow. | Nurse manager, assistant nurse managers to cover all shifts, multiple charge nurses. | Face to face,  Hospital emergency department | 6 weeks weekly meeting | No | NA |
| McKinley et al. (2007)  (Ref 19) | No name.  To reduce the risks of falling and the number of falling events. | NA | Review and analysis the baseline data, team consensus conference. | Fall Prevention Program team | Face to face, clinical wards  Hospital | NA | No | NA |
| Ostaszkiewicz et al. (2021)  (Ref 20) | No name  To co-design and pilot test a best practice model of continence care and knowledge translation resources for use in  Australian residential aged care homes | The best practice model of continence care and knowledge translation resources | A scoping review,  identification and review of frameworks and policy, online survey, qualitative interviews, workshops. | Registered Nurses, Enrolled Nurses, Personal Care Workers and one Nurse Unit Manager | Face to face,  Residential aged care homes | NA | No | NA |
| Parchment & Stinson (2020)  (Ref 21) | No name.  To identify and holistically address the health care needs of trafficked victims throughout the risk continuum. | NA | System-wide assessment. | Clinical nurses, nurse leaders | Face to face  NA | 3 months | No | NA |
| Britt Pipe (2007)  (Ref 22) | No name.  To align the processes of EBP and theory-driven  care. | Rosswum and Larrabee framework. | Survey, literature review, committee meetings. | Hospital staff | Face to face,  Hospital clinic | NA | No | NA |
| Robbins et al. (2017)  (Ref 23) | No name.  To reduce the incidence of nursing turnover within this demanding healthcare environment and ensure optimal patient care. | NA | Form a working group, systematic review, journal club team meetings, developed a nurse transition program. | VNIP trainer and Preceptor (qualified staff nurses) | Face to face,  Hospital clinic | 3-day “train-the-trainer” course for stakeholders, 8-12 weekly training, 6 months mentoring. | No | Receiving unique name badge and pin, on-going training and updates, workload included in the evaluation. |
| Salvador & Howell (2010)  (Ref 24) | No name.  To guide interventions  aimed at reducing symptom severity and distress in stem cell transplant patients  undergoing high-dose chemotherapy. | NA | Internal evidence, professional practice knowledge, and relevant theories  and models were synthesized. | A master-prepared staff nurse, with assistance from the Chair of the Oncology Nursing Research. | NA  Hospital | NA | No | NA |
| Stacey et al. (2019)  (Ref 25) | No name.  To enhance quality of cancer symptom support by homecare nurses | Knowledge to Action Framework | Survey, interviews, adapted guides for local use, implemented with interventions to address barriers, monitored use. | Registered nurses and registered practical  nurses. | Face-to- face training sessions, online tutorials.  Nursing agencies | NA | No | NA |
| Sving et al. (2020)  (Ref 26) | Evidence-based intervention pressure ulcer prevention.  To prevent pressure ulcer. | NA | Multifaceted, unit-tailored intervention developed by research group. | Registered nurse, dietician, physiotherapist, occupational therapist. | Face to face,  Hospital cinic | 1-day educational seminar, quality measurements per month. | After two years the role of the external facilitator changed | NA |
| Tafelmeyer et al. (2017)  (Ref 27) | No name.  To identify processes, outcomes, and lessons learned from designing a new evidence-based unit. | NA | Survey, sensor technic used for data collection to identify the evidence for the unit design. | Core team of nurse leaders | Face to face,  Hospital clinic | Weekly meetings,  weekly walk-through,  monthly council meetings,  quarterly staff meetings | No | NA |
| Thomas & Donohue-Porter (2012)  (Ref 28) | No name.  To improve intershift handoffs in a multihospital setting | Three theoretical frameworks of change, communication, and caring through dialogue | Design a framework, Didactic and interactive session. | Academic member from the system’s research and evidence-based practice council ) | Face to face,  Hospital clinic | The education session is two weeks. | No | NA |
| Thomas et al. (2020)  (Ref 29) | No name.  To reduce both the incidence and prevalence of hospital-acquired pressure injuries (HAPIs) by utilizing an evidence-based pressure injury prevention bundle. | NA | NA | NA | Face to face,  Hospital clinic | NA | No | NA |
| Van Orne (2021)  (Ref 30) | No name.  To reduce the need for invasive constipation treatment and to improve nurse satisfaction. | NA | Nurse satisfaction survey, forming a multidisciplinary team, review the guidelines, develop a plan, flash skills education. | RCU CNL, unit pharmacist, a pediatric nurse practitioner, the clinical educator, an occupational therapist, and the Director of Nursing Research and Evidence Based Practice | Face to face,  Hospital clinic | 8 sessions education in 4-day period | No | NA |
| Yurumezoglu & Kocaman (2012)  (Ref 31) | No name.  To increase the levels of job satisfaction and organizational commitment, minimize the intent to leave among the nurses in the hospital. | NA | Booklet.  Searching the literature, selecting and integrating the evidence, face-to-face interactive group meetings. | Nurse managers | Face to face,  Hospital | 10 weeks, 90 mins weekly meetings | No | NA |

NA= Information not available
