# Supplementary material for: The impact of evidence-based nursing leadership in healthcare settings: a mixed methods systematic review
Source: BMC Nurs. 2024 Jul 3;23:452. doi: 10.1186/s12912-024-02096-4 (PMC11221094; doi:10.1186/s12912-024-02096-4)
Supplement: Supplementary file 6 — Supplementary Material 6 [file 12912_2024_2096_MOESM6_ESM.docx]

**Additional file 6: Narrative description of the intervention or program components**

Out of 31 studies, nine studies had a name for the specific intervention or program, such as Group Clinical Supervision (GCS) (ref 1); Internship program (ref 3), Digging for Dinosaurus (ref 4), CUSP (ref 10), Quality Improvement Project, QI (ref 11), Nursing shared governance (ref 14), CO Strategies (ref 15), ED Leadership Model (ref 17), and Evidence-based intervention for pressure ulcer prevention (see Supplementary material 5 for description of interventions) (ref 26).

Six (n=6) (19%) studies used a specific framework to guide the study project. In one study (ref 1), the Clinical Nursing Leadership Learning and Action Process (CLINLAP) model was used [1]. The model views strategic nursing leadership and learning as essential prerequisites for the development of nursing practice, within the complex social and organizational milieu of the health and social care sectors. Galiano (ref 6) used Donabedian’s Quality Model [2] as a theoretical approach, while Ostaszkiewicz (ref 20) used the best practice model of continence care and knowledge translation resources for use in Australian residential aged care homes. Further, Pipe (ref 22) used the model by Rosswum & Larrabee [3] for guiding nurses through a systematic process for the change to EBP using a solid grounding in change theory and the principles of research utilization and the use of standardized nomenclature. In addition, the Knowledge to Action Framework was used in Stacey’s study (ref 25). In Thomas’s study (ref 28), three theoretical frameworks of change, communication, and caring through dialogue guided the design and implementation of evidence-based strategies that ultimately allow for knowledge translation (see Supplementary material 5 for description of interventions) (ref 23, ref 25, ref 26).

Five studies (16%) identified material used to support the intervention. These included a booklet, a checklist, and a model for the implementation (n=5) (16%) (ref 1, ref 2, ref 4, ref 17, ref 31). The provider of the program varied. Typically, there was a multi-professional team, which was formed for the study purposes (n=11) (35%) (ref 2, ref 5, ref 10, ref 11, ref 14, ref 15, ref 19, ref 23, ref 27, ref 29, ref 30). The providers were also clinical nurses (ref 2, ref 4), nurse managers (ref 27, ref 31), clinical managers (ref 7, ref 12), or patient care managers (ref 17). Nurses and researchers with EBP backgrounds (ref 3, ref 6, ref 28) and specialized nurses were included in five studies (16%) (ref 8, ref 12, ref 23, ref 26, ref 30). Regarding models of delivery, most were delivered face to face (ref 1, ref 3, ref 5, ref 6, ref 9, ref 10, ref 11, ref 12, ref 13, ref 14, ref 15, ref 16, ref 17, ref 18, ref 19, ref 20, ref 21, ref 22, ref 23, ref 24, ref 26, ref 27, ref 28, ref 29, ref 30, ref 31). Some programs included a combination of face to face and telephone delivery (ref 7,8) and some were delivered both face to face and online (ref 4, ref 25). Typically, interventions or projects were conducted in hospital settings (ref 1, ref 2, ref 3, ref 4, ref 5, ref 6, ref 10, ref 11, ref 12, ref 13, ref 14, ref 15, ref 16, ref 17, ref 18, ref 19, ref 22, ref 23, ref 24, ref 26, ref 27, ref 28, ref 29, ref 30, ref 31). Home and community healthcare organizations (ref 7), homecare organizations (ref 8, ref 9), nursing agencies (ref 25) and aged care homes (ref 20) were used (see Supplementary material 5 for description of interventions).

Fifteen studies (48%) reported the dose of the intervention. The total lengths of the program varied; they were 4 days (ref 30), 6 weeks (ref 18), 10 weeks (ref 31), 12 weeks (ref 8,21), 16–20 weeks (ref 13), 20 weeks (ref 5,9), 33–37 weeks (ref 23) to 18–24 months (ref 3), The frequency of the intervention varied from weekly (ref 1, ref 18, ref 31) to monthly (ref 6). Some studies included a description of a mixed frequency depending on the program content (ref 23, ref 27), and others did not clearly mention the frequency. The timing of the individual sessions varied as well, depending on the type of program delivery, including 90 minutes sessions (ref 1, ref 31), 45 course hours (ref 6), one six-hour workshop and three telephone conferences from 10 to 30 minutes (ref 8, ref 9), a five-hour workshop (ref 9), a one-day seminar (ref 26), a three-day course (ref 23), three classroom days, one attendance day, eight workdays (ref 3), and a two-week education session (ref 28). Five studies (16%) had modified their programs (ref 1, ref 3, ref 7, ref 8, ref 31). Two studies (6%) evaluated the intervention deliverability and fidelity, and one study mentioned that they had adopted several strategies to promote adherence to the program (see Supplementary material 5 for description of interventions) (ref 23).
