# Supplementary material for: The impact of evidence-based nursing leadership in healthcare settings: a mixed methods systematic review
Source: BMC Nurs. 2024 Jul 3;23:452. doi: 10.1186/s12912-024-02096-4 (PMC11221094; doi:10.1186/s12912-024-02096-4)
Supplement: Supplementary file 7 — Supplementary Material 7 [file 12912_2024_2096_MOESM7_ESM.docx]

**Additional file 7: Leadership problems**

| **Author(s)**  **(year)**  **(Ref #)** | **Leadership problem to be solved** |
| --- | --- |
| Alleyne & Jumaa (2007)  (Ref 1) | Facilitating primary care nurses (District Nurse Team Leaders) to link management and leadership theories with clinical practice and improving the quality of the service provided to their patients. |
| Busbee et al. (2020 a,b)  (Ref 2) | Indwelling catheter placement without medical necessity, lack of alternative device use, broken sterile technique during insertion, improper maintenance, and unnecessary catheter longevity. Addressing escalating catheter associated urinary tract infection (CAUTI) rates, leading that the organization was not meeting goals. |
| Cullen & Titler (2004)  (Ref 3) | Implementing evidence-based practice is a complex process that requires support for nurses to make it a reality in care delivery. |
| Davidson & Brown (2014)  (Ref 4) | Encouraging nurses to question, change practice, and engage them to EBP. |
| DeLeskey (2009)  (Ref 5) | Are EBP used in prevention/management of post-operative nausea and vomiting? |
| Galiano et al. (2020)  (Ref 6) | How to promote implementation of EBP model |
| Gifford et al. (2011)  (Ref 7) | How to facilitate nurses use of guideline recommendations for diabetic foot ulcers |
| Gifford et al. (2013)  (Ref 8) | How to evaluate the influence of a leadership intervention on  nurses’ use of guideline recommendations in home care nursing. |
| Gifford et al. (2014)  (Ref 9) | How to support nurse managers and clinical leaders Evidence-informed decision making (EIDM) |
| Hester et al. (2016)  (Ref 10) | Reducing catheter-associated urinary tract infections (CAUTI) by revising urinary catheter management protocol |
| Hoke et al. (2016)  (Ref 11) | Postoperative urinary retention (POUR) is a common complication in postoperative patients, especially in patients undergoing spinal surgery. |
| Hsieh et al. (2016)  (Ref 12) | How to increase the effectiveness of and satisfaction with an electronic focus charting system, in order to decrease nurse burden and time spent in documentation. |
| Kidd et al. (2020)  (Ref 13) | How to ensure recruiting, staffing, and retaining nurses for the optimal  provision of cancer care into the future. |
| Kneflin et al. (2016)  (Ref 14) | Unwarranted variations in bathing practices across settings; whether bathing standardisation could address the recent increase in central line-associated bloodstream infections. |
| Laws et al. (2013)  (Ref 15) | The evidence is inconclusive as to whether better patient outcomes for behavioral issues in acute care are achieved using constant pattient observation; finding alternatives to reduce the use of sitters and constant observation of patients, while maintaining or improving patient safety. |
| McAllen et al. (2018)  (Ref 16) | Teaching hospital identified concerns about fall rates and patient and nurse satisfaction scores. |
| McDonough & Pemberton (2013)  (Ref 17) | Employee dissatisfaction with leadership, leadership turnover, inability of the nurse managers to properly manage over 200 employees. |
| McFarlan et al. (2019)  (Ref 18) | How to address the areas receiving low scores in consumer satisfaction survey: responsiveness to concerns and complaints during hospitalization, degree to which hospital staff worked as a team, likelihood to recommend, overall rating, staff identifying themselves to patients. |
| McKinley et al. (2007)  (Ref 19) | How to reduce falls in hospital |
| Ostaszkiewicz et al. (2021)  (Ref 20) | How to promote good continence care and knowledge translation resources for use in residential aged care homes. |
| Parchment & Stinson (2020)  (Ref 21) | Absence of standardized electronic human trafficking (HT) screening tools, resources to support trafficked victims, a consistent system-wide policy,  and trauma-informed HT education for nurses and other health care providers. |
| Britt Pipe (2007)  (Ref 22) | How to convey the importance of both evidence-based  practice (EBP) and theory-driven care in ensuring patient safety and optimizing outcomes. |
| Robbins et al. (2017)  (Ref 23) | Lack of a structured transition and training program was recognized as a contributing factor for nursing dissatisfaction and increased turnover. |
| Salvador & Howell (2010)  (Ref 24) | How to reduce symptom severity and distress (i.e. oral mucositis) in stem cell transplant patients undergoing high-dose chemotherapy. |
| Stacey et al. (2019)  (Ref 25) | Symptoms experienced by clients with cancer often occur at home and can become life-threatening, posing serious safety concerns. |
| Sving et al. (2020)  (Ref 26) | How to decrease high rates of pressure ulcers in hospital settings |
| Tafelmeyer et al. (2017)  (Ref 27) | How to design new hospital unit that would have a positive impact on patient outcomes, workflow, teamwork, and satisfaction. |
| Thomas & Donohue-Porter (2012)  (Ref 28) | Ineffective handoffs have been identified as a barrier to patient safety and quality and as a key area for improvement. |
| Thomas et al. (2020)  (Ref 29) | How to reduce incidence and prevalence of hospital-acquired pressure injuries. |
| Van Orne (2021)  (Ref 30) | Nurses felt their care was limited with few resources available to treat patients experiencing constipation. |
| Yurumezoglu & Kocaman (2012)  (Ref 31) | Preventing nurses from leaving the organization. |
