# Supplementary material for: The impact of evidence-based nursing leadership in healthcare settings: a mixed methods systematic review
Source: BMC Nurs. 2024 Jul 3;23:452. doi: 10.1186/s12912-024-02096-4 (PMC11221094; doi:10.1186/s12912-024-02096-4)
Supplement: Supplementary file 8 — Supplementary Material 8 [file 12912_2024_2096_MOESM8_ESM.docx]

**Additional file 8: Main features of evidence-based Leadership**

| **Author(s)**  **(year)**  **(Ref #)** | **Purpose** | **Organisational evidence or data collected** | **Scientific evidence identified and critically appraised** | **Views of stakeholders considered** | **Critical appraisal conducted** |
| --- | --- | --- | --- | --- | --- |
| Alleyne & Jumaa (2007)  (Ref 1) | To identify, create and evaluate effective processes for collaborative working so that the primary care nurses’ capacity for clinical/managerial decision-making could be improved to enhance the quality of care. | ‘Service issue analysis to come as close as possible to agree on specific goals.’ No further details.  Appraisal: No | NA  Appraisal: No | **‘**Focus on agreement by  stakeholders to implement  continuous quality service within an open relationship’s environment.’  Appraisal: No | No |
| Busbee et al. (2020 a,b)  (Ref 2) | To improve standardization of products, insertion techniques, and the development of nurse driven standing orders for removal to eradicate system wide CAUTI. | Following in-depth review of CAUTI cases and analysis of the data. No further details.  Appraisal: No. | NA  Appraisal: No | **S**teering committee formed by a Chief Nursing Officer and Clinical Practice Specialists.  Appraisal: No | No |
| Cullen & Titler (2004)  (Ref 3) | To promote implementing and evaluating a clinically relevant evidence-based practice change using internship for staff nurses. | If the literature did not provide information or it was not clear, community survey from an organization was done. Key stakeholders were interviewed why the practice is the way it is.  Appraisal: No | Research evidence collected.  Appraisal: The interns and teams reviewed the research evidence.  Appraisal: No | Experts and stakeholders sought to facilitate project success.  Appraisal: No | No |
| Davidson & Brown (2014)  (Ref 4) | To explore nurses’ willingness to question and change  practice. | Current practice reviewed through walk rounds, case reviews, interviews with staff; a gap analysis to determine what should be done vs. what is being done; history about why the practice is the way it is; policies, procedures, guidelines identified that need to change.  Appraisal: No | Question or PICO, keywords; present them to the librarian to refine and assist with search; sites and databased were provided; summary findings using ‘elevator speech’.  Appraisal: Interns and teams critiqued the evidence. | Find out the original authors and include them as stakeholders, interviews the history of the practice, ask the committees and members for approving the practice change.  Appraisal: No | Yes |
| DeLeskey (2009)  (Ref 5) | To bring research evidence for the management of post-operative nausea and vomiting into clinical practice. | Retrospective chart reviews.  Appraisal: No | NA  Appraisal: No | Stakeholder team met to determine the best strategy for change management, the area that fell short of the evidence-based criteria were discussed, strategies to address those areas were planned.  Appraisal: No | No |
| Galiano et al. (2020)  (Ref 6) | To describe an implementation programme for an evidence-based practice (EBP) model in a New Chilean hospital and to analyse the programme evaluation results. | Hospital's mission and vision statements vs. research culture established; the proportion of nursing alumni with formal EBP training.  Appraisal: No | Literature review performed (year 2000-): Wiley Online Library, Elsevier, CINAHL, Health Source: Nursing/ Academic Edition, PubMed, Cochrane Library.  Appraisal: No | NA  Appraisal: No | No |
| Gifford et al. (2011)  (Ref 7) | To describe the planning and evaluation of a leadership intervention to facilitate nurses’ use of guideline recommendations for diabetic foot ulcers in home health care. | Chart audit conducted for baseline (eight items in RNAO clinical guideline), which indicated a gap between guideline recommendations for practice and actual care.  Appraisal: No | The Canadian Diabetes Association (2008) and the RNAO (2005) clinical practice guidelines recommendations reviewed.  Appraisal: No | Interviews with nurse managers and clinical leaders (n=15), 1 workshop, 3 three follow-up teleconferences  Appraisal: No | No |
| Gifford et al. (2013)  (Ref 8) | To evaluate the influence of a leadership intervention on  nurses’ use of guideline recommendations in home care nursing. | NA  Critical appraisal: No | NA  Critical appraisal: No | NA  Critical appraisal: No | No |
| Gifford et al. (2014)  (Ref 9) | To field test and evaluate a series of organizational strategies to promote evidence-informed decision making (EIDM) by nurse managers and clinical leaders in home healthcare | Evidence facilitators worked with participants to identify priority problems.  Appraisal: No | Evidence facilitators worked with participants to access, appraise, and adapt the research evidence to the organizational context.  Appraisal: No | NA  Appraisal: No | No |
| Hester et al. (2016)  (Ref 10) | To reduce catheter-associated urinary tract infection (CAUTI) | NA  Appraisal: No | An extensive literature review of evidence based best practices.  Appraisal: No | NA  Appraisal: No | No |
| Hoke et al. (2016)  (Ref 11) | To raise the post-anesthesia are unit (PACU) nursing staff’s awareness of the potential for postoperative urinary retention (POUR) among postoperative patients and to develop an updated nursing practice algorithm for the evaluation and management of POUR in spinal surgery patients. | Clinical observations collected.  Appraisal: No | Electronic search in CINAHL and PubMed to retrieve articles.  Appraisal: Four areas emerged were critically reviewed. | The literature review was discussed with colleagues.  Appraisal: No | Yes |
| Hsieh et al. (2016)  (Ref 12) | To review and revise the focuses in the electronic charting system and to develop new focuses for documentation of clinical pathways; evaluate the impact of these changes on time required for documentation and nurse satisfaction | Nurses’ time (hours) documenting patient care assessed.  Appraisal: No | The CNS reviewed nursing policies, guidelines, journal  articles, and textbooks.  Appraisal: No | The unit director and senior nursing staff reviewed the contents after discussion; the final version reviewed and approved by the Quality Control Commission of the Nursing Department.  Appraisal: No | No |
| Kidd et al. (2020)  (Ref 13) | To summarize an innovative initiative in oncology nurse workforce development that addresses current and future gaps and encompasses use of dedicated education units for student nurse rotation and a transition-to-practice residency program. | Review of institutional data including original pilot analysis and ongoing programmatic metrics (N=8 years).  Appraisal: No | Professional guidelines, published literature.  Appraisal: No | NA  Appraisal: No | No |
| Kneflin et al. (2016)  (Ref 14) | To describe shared governance in action through the example of one pediatric institution’s decision to institute daily chlorhexidine bathing.  To describe the use of shared governance to make a hospital-wide practice change that positively impacted patient outcomes and strengthened the shared governance process. | The Nursing Professional  Practice Council (NPPC)  members realised two main themes among the bathing referrals: (1) unwarranted variations in bathing practices across settings and (2) the incidence of central line associated blood stream infections (CLABSIs) had been increasing.  Appraisal: No | PubMed search conducted.  Appraisal: Search results critically appraised in table. | Physicians were met to seek their feedback and support by Nursing Profession Practice Council, Nursing Profession Education Council, Nursing Profession Inquiry Council, Nursing Profession Coordinating Council, Patient Care Governance Council, NPPC Leadership.  Appraisal: No | Yes |
| Laws et al. (2013)  (Ref 15) | To design and implement proactive strategies to identify patients at risk for behavioral issues; to identify high-risk patients from a confusion and delirium perspective and to assess and develop guidelines for sitter usage as a mechanism of constant observation (CO) | The PI team partnered with the nursing staff to identify alternative CO strategies for other than sitter utilization as a 1^st^ option in consideration of patient safety. In 2011, fulltime employee use for sitters in the practice setting was 7.1 or 284 hours.  Appraisal: No | A review of the literature.  Appraisal: No | NA  Appraisal: No | No |
| McAllen et al. (2018)  (Ref 16) | To increase patient safety and patient and nurse satisfaction using patient bedside report | The team completed  a gap analysis to determine evidence-based best practices for shift report compared to the current practice; patient  satisfaction consistently scored below the target range of 90%; nursing communication scores below 85%; nurse satisfaction scores 69.7%; organizational assessment completed using SWOT.  Appraisal: No | A team of nursing administrators, directors, staff nurses, and a patient representative reviewed the literature and made recommendations for practice changes.  Appraisal: No | Staff consensus sought.  Appraisal: No | No |
| McDonough & Pemberton (2013)  (Ref 17) | Increasing volumes, increasing numbers of left without being seen (LWBS) patients, falling patient satisfaction numbers and a staff dissatisfied with ED leadership. | An employee opinion survey indicated significant dissatisfaction with emergency department (ED) leadership: employee satisfaction dropped by 41% from 2007 to 2009.  Appraisal: No | NA  Appraisal: No | NA  Appraisal: No | No |
| McFarlan et al. (2019)  (Ref 18) | To improve patient experience in the ED setting. | The group review of the baseline data was conducted.  Appraisal: No | The scholarly literature reviewed.  Appraisal: No | Process owners met weekly with teams of stakeholders to conduct random audits, to offer support and feedback.  Appraisal: No | No |
| McKinley et al. (2007)  (Ref 19) | The outcomes of a quality management initiative to reduce falls to illustrate the nexus between research and quality improvement in clinical practice. | Baseline data of falls were recorded and analysed to identify the extent of the problem.  Appraisal: No | Formal post-fall assessment protocol introduced.  Appraisal: No | The FPP (falls prevention program) team discussions provided the impetus for a number of management decisions.  Appraisal: No | No |
| Ostaszkiewicz et al. (2021)  (Ref 20) | To co-design and pilot test a best practice model of continence care and knowledge translation resources for use in Australian residential aged care homes (RACH). | NA  Appraisal: No | A scoping review of literature, identification and review of frameworks and policy documents about aged care, and the Aged Care Quality  Standards.  Appraisal: No | Two co-design workshops with 18 residential aged care stakeholders were organised to explore their perspectives about factors to include in a model prototype.  Appraisal: No | No |
| Parchment & Stinson (2020)  (Ref 21) | To ensure the safety and well-being of human trafficked victims and their teams. | NA  Appraisal: No | In collaboration with the librarian, a literature search was conducted.  Appraisal: No | Taskforce members met with regulatory representatives, nurse leaders, and other stakeholders.  Appraisal: No | No |
| Britt Pipe (2007)  (Ref 22) | To understand how nursing leadership convey the importance of evidence-  based practice and theory-driven care in ensuring patient safety and optimizing outcomes. | Internal data collected about current practice and compared with external data.  Appraisal: No | Literature review conducted.  Appraisal: No | Nursing leaders and staff nurses as the key stakeholders to discuss the need for a nursing model.  Appraisal: No | No |
| Robbins et al. (2017)  (Ref 23) | To implement an evidence-based transition to practice program specific to the burn specialty. | In 2011, the center experienced a nursing turnover of 33.6%.  Appraisal: No | Systematic review conducted.  Appraisal: Journal club team meetings to grade the level and quality of evidence. | NA  Appraisal: No | Yes |
| Salvador & Howell (2010)  (Ref 24) | To describe unit experience of reducing symptom severity and distress in stem cell transplant patients undergoing high-dose chemotherapy. | Staff nurse identified oral mucositis as a priority clinical problem that warranted change in their practice.  Appraisal: No | Internal evidence, professional practice knowledge, relevant theories and models synthesized.  Appraisal: No | Critical inputs from clinical educators, physicians, nutritionists, pharmacists, and nurse manager.  Appraisal: No | No |
| Stacey et al. (2019)  (Ref 25) | To evaluate implementation of evidence-informed symptom practice guides to enhance quality of cancer symptom support by homecare nurses. | Barriers to using COSTaRS practice guides assessed using interviews and a survey; a retrospective chart audit of documentation.  Appraisal: No | Individual studies  synthesized with systematic reviews or clinical practice  guidelines reviewed.  Appraisal: No | Knowledge users, leaders from the homecare authority, the vice-president clinical care, director of program development, an experienced family member were on the team.  Appraisal: No | No |
| Sving et al. (2020)  (Ref 26) | To assess sustainability of an intervention used to implement pressure ulcer prevention. | Managers in a county  experiencing a high prevalence of pressure ulcers (22%).  Appraisal: No | NA  Appraisal: No | Implementation team (nurse, dietician, physiotherapist, occupational therapist) to support the implementation of evidence-based prevention measures.  Appraisal: No | No |
| Tafelmeyer et al. (2017)  (Ref 27) | To designing a new evidence-based unit to impact patient and family outcomes. | NA  Appraisal: No | Literature search  Appraisal: Critically appraised relevant evidence. | NA  Appraisal: No | Yes |
| Thomas & Donohue-Porter (2012)  (Ref 28) | To implement a pilot for intershift handoff to test the improvements, | The degree and type of patient and family involvement in handsoff were not consistent; a variety of reporting templates were in use.  Appraisal: No | The literature review conducted.  Appraisal: No | Academic member from the  system’s research and evidence-based practice council, representatives from the 8 hospitals volunteering for the pilot implementation.  Appraisal: No | No |
| Thomas et al. (2020)  (Ref 29) | To reduce the incidence and prevalence of HAPIs by utilizing an evidence-based pressure injury prevention bundle. | A 531-bed acute care hospital has an HAPI prevalence of 5.5%; a gap analysis conducted.  Appraisal: No | Literature review  Appraisal: No | Nursing leadership and multidisciplinary teams supported the program.  Appraisal: No | No |
| Van Orne (2021)  (Ref 30) | How to reduce the need for invasive constipation treatment (suppository or enema), and to improve RCU nurse satisfaction with their ability to autonomously perform their job with accessible and evidence based tools to prevent and treat constipation. | Nurse satisfaction survey revealed that RCU clinical nurses felt their scope of practice was restricted by not having the resources needed; frustration with their lack of autonomy.  Appraisal: No | Review of literature.  Appraisal: No | Key stakeholders joined the team to provide relevant clinical knowledge and buy-in to ensure the successful implementation.  Appraisal: No | No |
| Yurumezoglu & Kocaman (2012)  (Ref 31) | To evaluate the effects of nurse managers’ use of evidence in managerial decisions and staff  nurses’ attitudes towards their jobs and organizations. | No systematic hospital-speciﬁc source of data on the levels of job satisfaction and organizational commitment used.  Appraisal: No | PubMed, Cochrane, and  EMBASE databases were searched, two journals were reviewed manually.  Appraisal: No | A summary of the findings and suggestions of the studies were discussed at 90 min, weekly meetings by 11 charge nurses and a nurse executive director over a 10 week period; charge nurses and the nurse executive director were consulted.  Appraisal: No | No |
