# Supplementary material for: The impact of evidence-based nursing leadership in healthcare settings: a mixed methods systematic review
Source: BMC Nurs. 2024 Jul 3;23:452. doi: 10.1186/s12912-024-02096-4 (PMC11221094; doi:10.1186/s12912-024-02096-4)
Supplement: Supplementary file 9 — Supplementary Material 9 [file 12912_2024_2096_MOESM9_ESM.docx]

**Additional file 9: Perceived effects of evidence-based leadership**

| **Author(s)**  **(year)**  **(Ref #)** | **Nurses’ performance** | **Organisational outcomes** | **Clinical outcomes** |
| --- | --- | --- | --- |
| Alleyne & Jumaa (2007)  (Ref 1) | Management and leadership interventions and approaches significantly influenced the participants’ capacity to improve the quality of services provided to their patients. Various techniques, tools, methods and frameworks used increased participants’ confidence to perform. | A structured approach maked implementing change more practical and manageable and getting agreement to do things differently much easier. | NA |
| Busbee et al. (2020 a,b)  (Ref 2) | NA | NA | NA |
| Cullen & Titler (2004)  (Ref 3) | Interns were motivated by a desire to change practice and their self-esteem was improved. | The projects gave learning opportunities and enriched staff nurse.  -Evidence-based practice is time-consuming, pays non-patient care time.  -Causes challenges in teamwork. | NA |
| Davidson & Brown (2014)  (Ref 4) | NA | NA | NA |
| DeLeskey (2009)  (Ref 5) | NA | NA | NA |
| Galiano et al. (2020)  (Ref 6) | NA | NA | NA |
| Gifford et al. (2011)  (Ref 7) | Intervention influenced of guideline implementation; increased motivation and enthusiasm while keeping the guideline implementation a priority. | Helped mentor and staff in their efforts to make change.  Intervention validated the importance of working together as a team, reinforced each member’s unique role in supporting practice change; helped mentor and staff in their efforts to make change.  The intervention assisted to make the connection between the guideline recommendation and patient outcomes. | NA |
| Gifford et al. (2013)  (Ref 8) | In experimental group, participants were more engaged with staff, encouraging them to adopt the new practices and recognizing their efforts to change; they took a lead role in the implementation process, being visible, communicating with staff, and were more involved in the planning and decision-making related to implementation. In experimental group, participants identified using different change-oriented behaviors to initiate, promote, and implement change | NA | NA |
| Gifford et al. (2014)  (Ref 9) | NA | NA | NA |
| Hester et al. (2016)  (Ref 10) | NA | NA | NA |
| Hoke et al. (2016)  (Ref 11) | NA | NA | NA |
| Hsieh et al. (2016)  (Ref 12) | NA | NA | NA |
| Kidd et al. (2020)  (Ref 13) | NA | NA | NA |
| Kneflin et al. (2016)  (Ref 14) | NA | NA | NA |
| Laws et al. (2013)  (Ref 15) | NA | Organization and nurse leaders have realized a more appropriate use of sitters, CO, and CO alternatives while still providing a safe environment patients. | NA |
| McAllen et al. (2018)  (Ref 16) | Time efficiency:  “...printed would make it more efficient” | NA | Good for the patient:  “...but it is worth it”  “...is worth it for the patient” “...better”  “... it is good for the patient”  “It is good for the patient” “...positive impact for the patient.  Patient safety:  “...improves time, and overall patient safety”  “... we were able to address a neuro status that had changed from report to the bedside” “I like it and we may want to expand and do NIH at bedside at BSR”  “...it is nice to have eyes on all my patients and we can check orders are done.” |
| McDonough & Pemberton (2013)  (Ref 17) | NA | NA | NA |
| McFarlan et al. (2019)  (Ref 18) | NA | NA | NA |
| McKinley et al. (2007)  (Ref 19) | NA | NA | NA |
| Ostaszkiewicz et al. (2021)  (Ref 20) | Staff­ knowledge, skills, and education. | Adequate resources.  Practical assistance to optimise a resident’s continence. Importance of the components of the draft mode (14 residential aged care stakeholders); person-centred continence care. | NA |
| Parchment & Stinson (2020)  (Ref 21) | The majority of nurses reported an increase in knowledge comprehension and application of the subject matter. | NA | NA |
| Britt Pipe (2007)  (Ref 22) | Positive and enthusiastic about the systematic translation of theory into practice; appreciation of theory-driven practice with a language that empowers them to speak about what they do and who they are with patients; multiple examples when caring behaviors (intentional presence, listening, developing empathy) had led to advocacy for health and safety. | NA | NA |
| Robbins et al. (2017)  (Ref 23) | NA | NA | NA |
| Salvador & Howell (2010)  (Ref 24) | NA | NA | NA |
| Stacey et al. (2019)  (Ref 25) | One nurse who used a COSTaRS practice guide when awoken in the night said she felt more relieved that she had handled the client symptom appropriately and was able to return to sleep without revisiting the call. | NA | NA |
| Sving et al. (2020)  (Ref 26) | Systematic work increased nurses’ awareness of the patients at risk of developing pressure ulcers and led to the development of new pressure ulcer prevention routines. |  | NA |
| Tafelmeyer et al. (2017)  (Ref 27) | NA | NA | NA |
| Thomas & Donohue-Porter (2012)  (Ref 28) | Increase in knowledge about patient priorities and an opportunity for questions about their accountability to the plan of care for new nurses; new graduates reported feeling empowered. | Initial feedback was unfavorable, nurses experienced discomfort at sharing sensitive information at the bedside, concerns over some extended time being taken for report, and problems when multiple patients were on isolation. | “This will not work and is a waste of time on the unit” was the opinion of several staff members who said they would not hinder the implementation but eventually would be seen as correct. |
| Thomas et al. (2020)  (Ref 29) | NA | NA | NA |
| Van Orne (2021)  (Ref 30) | NA | NA | Narrative feedback speciﬁcally regarding the bowel management algorithm included 100% positive feedback; “It seems pretty common sense to have PRNs and guidance of when to use them, but it was never available before.” |
| Yurumezoglu & Kocaman (2012)  (Ref 31) | Nurse director: the study addressed an actual problem, therefore is very useful.  Charge nurses: nurses more willing to stay with the organization and there was an increased level of motivation after the implementation of evidence-based nursing management practices. | Nurse director: Fewer staff nurses than expected left the organization over the course of the study. | NA |
