# Supplementary material for: The impact of evidence-based nursing leadership in healthcare settings: a mixed methods systematic review
Source: BMC Nurs. 2024 Jul 3;23:452. doi: 10.1186/s12912-024-02096-4 (PMC11221094; doi:10.1186/s12912-024-02096-4)
Supplement: Supplementary file 10 — Supplementary Material 10 [file 12912_2024_2096_MOESM10_ESM.docx]

**Additional file 10: Measured effects of evidence-based leadership**

| **Author(s)**  **(year)**  **(Ref #)** | **Nurses’ performance** | **Organisational outcomes** | **Clinical outcomes** |
| --- | --- | --- | --- |
| Alleyne & Jumaa (2007)  (Ref 1) | NA | NA | NA |
| Busbee et al. (2020 a,b)  (Ref 2) | NA | Routine placement of indwelling urinary catheters declined; alternative device use increased; sterile technique during insertion improved. Product life cycle assessments demonstrated improvements related to indwelling catheter use and care. | The incidence of CAUTI decreased 27.8% (2015-2019). |
| Cullen & Titler (2004)  (Ref 3) | The process for using evidence-based practice (Mean 4.5, range 4–5; Helped to grow professionally (Mean 4.8, range 4–5); Understand how to use the Iowa Model to Promote Quality Care (Mean 4.5, range 4–5); Stimulated innovative thinking (Mean 4.8, (4–5); Gave knowledge needed to use evidence-based practice to answer clinical questions (Mean 4.5, range 4–5); Made it possible to complete an evidence-based practice project (Mean 4.3, range 3–5). | NA | NA |
| Davidson & Brown (2014)  (Ref 4) | NA | More involvement in organizational practice: 15 nurses reported 23 practice improvement opportunities; 14 projects changed practice; 4 were presented at conferences. | NA |
| DeLeskey (2009)  (Ref 5) | PONV risk factors are documented was being met 2.5% of the time, and criterion 2: PONV risk factors are communicated among anaesthesia and surgical staff, was not being met at all. Criteria 1 and 2 increased to 63% and 62% compliance, respectively. Criterion 3: PONV treatment is ordered as indicated and criterion 4: PONV prophylaxis is administered as indicated by risk factors, had 69% and 30% compliance before the change, and increased to 77% and 49% following change management. The final two criteria: assessment for PONV occurs and rescue treatment is initiated as needed were both at 100% following the project. Before change management, they were at 97% and 100%, respectively. | NA | Patients’ post-operative nausea and vomiting decreased from 18% at the start to 5% nausea and 0% vomiting at the end. |
| Galiano et al. (2020)  (Ref 6) | Nurses participated in 26 conferences (abstract acceptance rate 100%) 7 articles published, 1 rejected;  EBP knowledge: a statistically significant improvement (p = .038); those with formal EBP training showed a significant difference of 8.6% (0.6 points) in the average CPBE-19 score in knowledge, attitude and application in the last evaluation (p < .01); attitude towards EBP remained stable (p=.543); those who applied EBP decreased, no significant differences over the years (p=.879). | NA | NA |
| Gifford et al. (2011)  (Ref 7) | Identification of priority indicator and target outcomes (mean = 3.7), chart audit findings about the research/practice gap (mean = 3.5), discussions of barriers and leadership supports (mean = 3.5) and development of the leadership action plan (mean = 3.5) out of possible max value 4.  Top two items: identification of priority indicators and target outcomes (mean = 8.1) and development of leadership action plans (mean = 8) out of possible max value 10. | NA | NA |
| Gifford et al. (2013)  (Ref 8) | NA | No statistically significant difference between control and experimental groups in nurses’ assessment of diabetic foot ulcers. Five-item priority indicator score: a significantly higher mean, median, and distribution of scores between control and experimental groups (p < 0.05). Adjusted cluster effects with the observed intraclass correlation coefficient (ICC = 0.01): a higher mean score was confirmed in the experimental group (p = 0.008). | NA |
| Gifford et al. (2014)  (Ref 9) | NA | Four items (acquire, assess, adapt, and apply research findings) on the Canadian Foundation for Healthcare Improvement (CHSRF) survey displayed statistically significant increases after the intervention (P<0.5). The items reflected the organization’s capacity for participants to acquire and apply research findings in their decision making. | NA |
| Hester et al. (2016)  (Ref 10) | Use of the Urinary Catheter Management Algorithm (UCMA) was greater than 95%. | NA | CAUTI (Catheter-associated urinary tract infection) infections reduced to 0. |
| Hoke et al. (2016)  (Ref 11) | The number of indwelling urinary catheters inserted in the PACU was decreased, the PACU nursing staff was supporter in more frequent and appropriate use of intermittent catheterization in patients undergoing spinal surgery. | In the preintervention group, 19 indwelling urinary catheterizations were documented in 42 patient records; no use of intermittent catheterization was documented. In the postintervention group, seven indwelling urinary catheterizations were documented in 43 patient records; the use of intermittent catheterization for bladder volumes of more than 450 mL was documented in 11. No differences in intermittent catheterization or indwelling urinary catheter use were identified based on area of surgery (cervical or lumbar) or type of surgical procedure (laminectomy or fusion). |  |
| Hsieh et al. (2016)  (Ref 12) | Satisfaction with usability showed clinical significance after introduction of the intervention (mean T SD, 3.7 T 0.7 preimplementation vs 4.1 T 0.62 postimplementation). | The total time for documentation during a 1-week period decreased from 138.5 hours in the preimplementation group  to 55.8 hours postimplementation. Documentation time decreased significantly during the day shift (72.5 hours preimplementation vs 28.7 hours  postimplementation) and evening shift (61.9 vs 22.5 hours), but not during the night shift (4.1 vs 4.5 hours). | NA |
| Kidd et al. (2020)  (Ref 13) | NA | The number of nurse participants in presentations from increased steadily (5 in 2012 to over 70 in 2019). Staff professional development and revealed a 98% retention rate within those nurses hired to the nurse residency program; hire rates of 13% inpatient adult, and 87% in the outpatient department. | NA |
| Kneflin et al. (2016)  (Ref 14) | NA | NA | The incidence of CLABSIs at institution dropped following implementation of CHG bathing. Patients with central lines are at decreased risk for acquiring a central line-associated blood stream infection due. |
| Laws et al. (2013)  (Ref 15) | NA | 80% decrease in medical/surgical workplace injuries from combative patients (March 2011 to October 2012)  The PI team exceeded the year-to-date goal of reducing sitter use by 20% and 2.5 full-time employee. | Medical/surgical patient falls per 1000 patient-days 0.47 Falls per 1000 patient-days (August 2012 to October 2012). Medical center-wide patient falls with major injury or death per 1000 patient-days 0 Falls/1000 patient-days (June 2012 to October 2012) |
| McAllen et al. (2018)  (Ref 16) | **Compliance**: compliance rate of bedside shift report (BSR) 94% (n= 157).  **Satisfaction:** Nurses who reported having enough time for report significantly decreased (from 80% to 59.6%) after implementation of BSR (-2.668, p = 0.008); 70% (n = 45) of nurses believed that BSR increased the time it took to individually give and receive report; 39% reported concerns about patient confidentiality; 44% (n=29) expressed that BSR was inconvenient. | No statistically significant difference between mean time for report before and after implementation of BSR. | Patient falls decreased by 24% in the four months after BSR implementation; reduction in the number of falls: orthopedic unit (55.6%), neuroscience unit (16.9%), and the general surgery (6.9%); the general surgery unit had statistically significant improvement in patient satisfaction after implementation (p = 0.03) (the 8 questions) increased from average score 87.7% to 91.6%. HCAHPS showed improvement, but the changes were not statistically significant. |
| McDonough & Pemberton (2013)  (Ref 17) | Nurse survey (2009 -2010) showed significant changes in communication, management and overall job satisfaction; “My manager supports associate suggestions that are meant to correct existing problems” (306%), “I know what is expected of me in my job” (318%), “Communication between management and associate is more effective since the ED leadership structure changes” (214%), “I see management more often as a result of the ED leadership structure change” (315%), “Recent ED leadership structure change has helped me to have a better relationship with my manager” (150%). | 2009-2010 the walkout rate reduced; 2012-2013 nurse staff vacancies had been reduced to less than 2 FTEs from 16 | Patient satisfaction scores improved by 600% from 2009; by the fourth quarter of 2012, patient satisfaction scores had reached the 99th percentile; the percentage of patients who left without being seen was below 2%. |
| McFarlan et al. (2019)  (Ref 18) | Compliance improved (from 60% to over 90% . | NA | Patient experience scores (5 survey items) all  improved: (1) response to concerns/complaints during your stay; (2) degree to which  hospital staff worked as a team; (3) staff identified themselves to patients; (4) overall rating of institution; (5) likelihood to recommend. |
| McKinley et al. (2007)  (Ref 19) | NA | NA | Rate of falls 0.25% (2006), which is under the maximum national aggregate of 0.37%, and better than the peer group rating of  0.28%; a substantial reduction in the number of falls per bed day since the introduction of the FPP and a significant reduction in the number of patients experiencing multiple falls within one episode of hospitalization. |
| Ostaszkiewicz et al. (2021)  (Ref 20) | Pilot test revealed post-education improvements in 8-10 knowledge questions; 63% rated the education as ‘very helpful’ in applying the model to practice; 37% rated ‘somewhat helpful’ (177 residential aged care stakeholders as family carers and aged care staff­) | 100% agreement on the feasibility, appropriateness and acceptability of  the model and education program (177 residential aged care stakeholders). | NA |
| Parchment & Stinson (2020)  (Ref 21) | 90% (n =872) of nurses completed the HT education. | 46 764 patient encounters transpired, and all were screened; of those, 43 were identified as potential trafficked victims. | NA |
| Britt Pipe (2007)  (Ref 22) | NA | NA | NA |
| Robbins et al. (2017)  (Ref 23) | 26/30 (87%) completed the transition program, 3 (10%) did not complete, 1 (3%) received exception; 22 (76%) nurses achieved passing BKAT scores, 24 (93%) passed the Wound Care test; Tracking Progress Toward Independent Practice weekly evaluations averaged 52 and final ratings averaged 91 (n=25, p<0.0001). | Turn-over rate decreased (33.6% to 16.5%; p<0.1); nurses left due to career advancement (n=10, 56%), personal obligations or commitments (n=5, 28%), job dissatisfaction (n=1, 6%), inability to complete transition program (n=2, 11%). | NA |
| Salvador & Howell (2010)  (Ref 24) | NA | Aﬀorded a proactive approach to mucositis prevention with an evidence-based oral care guide. | Achieved a signiﬁcant reduction of mucositis severity/distress through a pilot randomized trial. |
| Stacey et al. (2019)  (Ref 25) | Higher confidence in symptom management (p < 0.01); evidence of COSTaRS practice guide use in 16%, 22% and 70% for each of the 3 agencies who audited charts (Cases C, B, E, respectively); nurses from 3 agencies self-reported use of COSTaRS practice guides (35%, 91%, 100%; Cases C, B, E respectively). | NA |  |
| Sving et al. (2020)  (Ref 26) | NA | Significant improvement the use of bed sliding sheets and turning schedules and the risk assessment documentation within 24 h of admission. | All patients a significant reduction in the prevalence (category 2–4) pressure ulcers at the long-term follow-up (P=0.021); difference in prevalence for the patients at risk of developing pressure ulcers not statistical significance; pressure ulcer prevention measures/patient increased at the long-term follow-up with a significant improvement (P <0.001); increase in the offloading of heels between baseline and short-term follow-up was sustained at the long-term follow-up. |
| Tafelmeyer et al. (2017)  (Ref 27) | A significant decrease was noted in the item nurses spent significantly more time in the clean utility room (P = .0), medication room (P = .037), Hallway (.074), and patient room (P = .0) on the new unit and significantly less time in the equipment room (P = .025), nurses’ station (P = .016), and off the unit (P = .044); more time was spent assessing, ambulating, and toileting patients on the new unit. | Two significant improvements in team work scores: charge nurses balanced workload (P = .047), and team members clearly communicated their expectations of others (P = .048); noise was 5.18 dB lower at the nursing station and 4.88 dB lower in the patient room or approximately 5 times lower in the new unit. | Falls: from 2.98 falls per 1000 patient days to 2.72 falls per 1000 patient days; MRSA transmissions: 6 transmissions but a significant increase in patient days, thus a lower transmission rate of 6.081 × 10^-4^ incidents per 1000 patient days.  Noise demonstrated a significant change with patients being more satisfied on the new unit (P = .007); ); noise was 4.88 lower in the patient room or approximately 5 times lower in the new unit.  Patient experience: cleanliness, nursing communication, and responsiveness of staff all improved on the new unit, but trends were not significant. |
| Thomas & Donohue-Porter (2012)  (Ref 28) | Nurse satisfaction: although the aggregated data for the 7 hospitals showed improvement for all indicators, the units showed differences in their improvement; nurses had adequate time for the inter shift report, appropriate information was being transferred, and relationships between shifts had improved. | NA | Patient satisfaction scores: Patient  satisfaction scores improved with the implementation of the bedside handoff process. |
| Thomas et al. (2020)  (Ref 29) | NA | NA | The prevalence rate of HAPI degreased: 2014 was 4%; 2015 the rate was 1.24%; 2016 was 0.43%; 2017 was 0.41%; 2018 was 0.44%, 2019 was 0.25%. Incidence rates 2016 was 0.85%; 2017 was 0.70%; 2018 was 0.45%; 2019 was 0.29%. |
| Van Orne (2021)  (Ref 30) | Staff satisfaction improved “How satisﬁed are you with the tools and resources available for you treat and prevent patient constipation?” (54%, n=17 vs. 92%, n = 35, p < 0.001). | Statistically signiﬁcant reduction in average rate of invasive medication administration between pre-intervention and post-intervention (p = 0.01). After excluding patients who received invasive constipation management medications within 24-h of admission to RCU for constipation present prior to admission, the rates were further reduced. |  |
| Yurumezoglu & Kocaman (2012)  (Ref 31) | A statistically significant differences in all categories of satisfaction between the baseline and both follow-up measurements (P < 0.05). | Organisational commitment: A statistically significant difference in normative commitment across measurements (P < 0.05). No statistically significant difference in the affective commitment, continuance commitment, and intent to leave (P > 0.05). | NA |
